# Potential effect of household contact management on childhood tuberculosis: a mathematical modelling study

**DOI:** 10.1016/S2214-109X(18)30401-7

**Published:** 2018-09-25

**Authors:** Peter J Dodd, Courtney M Yuen, Mercedes C Becerra, Paul Revill, Helen E Jenkins, James A Seddon

**Affiliations:** a School of Health and Related Research, University of Sheffield, Sheffield, UK; b Division of Global Health Equity, Brigham and Women's Hospital, Boston, MA, USA; c Department of Global Health and Social Medicine, Harvard Medical School, Boston, MA, USA; d Partners In Health, Boston, MA, USA; e Centre for Health Economics, University of York, York, UK; f Department of Biostatistics, Boston University School of Public Health, Boston, MA, USA; g Centre for International Child Health, Department of Paediatrics, Imperial College London, London, UK; h Desmond Tutu TB Centre, Department of Paediatrics and Child Health, Faculty of Medicine and Health Sciences, Stellenbosch University, Cape Town, South Africa

## Abstract

**Background:**

Tuberculosis is recognised as a major cause of morbidity and mortality in children, with most cases in children going undiagnosed and resulting in poor outcomes. Household contact management, which aims to identify children with active tuberculosis and to provide preventive therapy for those with HIV or those younger than 5 years, has long been recommended but has very poor coverage globally. New guidelines include widespread provision of preventive therapy to children with a positive tuberculin skin test (TST) who are older than 5 years.

**Methods:**

In this mathematical modelling study, we provide the first global and national estimates of the impact of moving from zero to full coverage of household contact management (with and without preventive therapy for TST-positive children older than 5 years). We assembled data on tuberculosis notifications, household structure, household contact co-prevalence of tuberculosis disease and infection, the efficacy of preventive therapy, and the natural history of childhood tuberculosis. We used a model to estimate households visited, children screened, and treatment courses given for active and latent tuberculosis. We calculated the numbers of tuberculosis cases, deaths, and life-years lost because of tuberculosis for each intervention scenario and country.

**Findings:**

We estimated that full implementation of household contact management would prevent 159 500 (75% uncertainty interval [UI] 147 000–170 900) cases of tuberculosis and 108 400 (75% UI 98 800–116 700) deaths in children younger than 15 years (representing the loss of 7 305 000 [75% UI 6 663 000–7 874 000] life-years). We estimated that preventing one child death from tuberculosis would require visiting 48 households, screening 77 children, giving 48 preventive therapy courses, and giving two tuberculosis treatments versus no household contact management.

**Interpretation:**

Household contact management could substantially reduce childhood disease and death caused by tuberculosis globally. Funding and research to optimise its implementation should be prioritised.

**Funding:**

UK Medical Research Council, US National Institutes of Health, Fulbright Commission, Janssen Global Public Health.

## Introduction

Tuberculosis is the leading infectious cause of mortality worldwide, affecting an estimated 1 million children in 2016, of whom an estimated 253 000 died in 2016.^1^ Tuberculosis is a top ten cause of global under-5 mortality,^2^ with most deaths each year occurring among the roughly half a million children who are never diagnosed or treated.^2^, ^3^ Younger children are more likely to develop severe forms of tuberculosis, such as tuberculous meningitis, that are often fatal or have long-term sequelae (eg, neurological deficits).^4^ These deaths and long-term effects have a substantial impact on families and society; these young children would otherwise have had many decades of productive life ahead of them. To reduce the burden of childhood tuberculosis disease and death, more children with tuberculosis need to be diagnosed and treated or prevented from becoming sick with tuberculosis in the first place. Preventing cases of tuberculosis is especially important in resource-limited settings, where the diagnosis of children with tuberculosis can be particularly challenging.

One of the most effective ways of identifying children with both tuberculosis infection and disease is through household contact investigations, because children living in the homes of adults with tuberculosis are at high risk of both infection and disease.^5^ Systematically assessing child household contacts can ensure that children with tuberculosis disease are diagnosed and treated early, and that children with tuberculosis infection or exposure are given preventive therapy to prevent them from becoming sick in the future. Since 2012, WHO guidance has recommended household contact investigations for patients with tuberculosis and preventive therapy for children younger than 5 years and people living with HIV.^6^ In 2018, this guidance was updated to include the option of preventive therapy for older contacts with a positive tuberculin skin test (TST) in settings with a high tuberculosis burden.^7^ Despite the fact that many national policies incorporate these recommendations,^8^, ^9^ major gaps exist in the implementation of household contact management (HCM).^10^, ^11^ Worldwide, only 13% of eligible children younger than 5 years are estimated to receive preventive therapy.^1^ Competing priorities, low awareness, infrastructural challenges, stigma, and inadequate access to care are all barriers.^10^ Until recently, HCM activities were not routinely included in monitoring and reporting systems; coverage of preventive therapy to children younger than 5 years who are household contacts of bacteriologically confirmed pulmonary tuberculosis has now been requested and reported by WHO since 2016.^1^

**Research in context**

**Evidence before this study**

We did a PubMed search in March, 2018, using the search terms “Tuberculosis” AND “Household” AND “Contact” AND “Child*” AND “Model*”. We found 65 articles, from which we identified two studies modelling the effect of household contact management of children. Morrison and colleagues and Fox and colleagues each conducted a systematic review and meta-analysis on the co-prevalence of tuberculosis disease and infection in household contacts and reported similar findings, which we used in our analysis. We used systematic reviews and meta-analyses on the efficacy of isoniazid preventive therapy in preventing tuberculosis in children who are HIV negative (Ayieko and colleagues) and HIV positive (Zunza and colleagues) in our analysis. Mandalakas and colleagues did a cost-effectiveness analysis of household contact activities focused on children younger than 5 years using South African cost data and found it to be a cost-effective intervention, but did not consider co-prevalent cases. Our analysis focused on hypothetical cohorts of household contacts and did not assess the numbers of household contacts. Yuen and colleagues estimated the number of child contacts expected from household contact activities, but not the effect of such activities on tuberculosis disease and death in children.

**Added value of this study**

Our study is the first to project the effect on childhood disease and death of tuberculosis household contact management among children, by bringing together models of contact numbers and of cohort outcomes for 217 countries and territories. We incorporated systematic review evidence on yields of household contact investigations in children, child-specific efficacy of preventive therapy and tuberculosis outcomes, and data on HIV and antiretroviral therapy. We also include the benefits of identifying co-prevalent tuberculosis disease in children and of preventing incident disease.

**Implications of all the available evidence**

Household contact activities for tuberculosis could have potentially prevented the loss of about 110 000 lives with 7 million years of life expectancy in 2016 in children younger than 15 years, with measures of demands on the health-care system that suggest excellent value for money. The global use of household contact management should be increased. Operational research and local cost-effectiveness analysis to inform implementation should be priorities.

Given the poor implementation of household contact investigations and preventive therapy despite universal acknowledgment of their importance, we aimed to quantify the potential effect that full use of these interventions could have. This knowledge would permit an analysis of the effect of HCM compared with other childhood tuberculosis measures or other measures to reduce child mortality. We brought together available data within a mathematical modelling framework to estimate the potential global reduction in childhood tuberculosis disease and death that child-targeted household contact interventions could achieve if taken to scale.

## Methods

### Study design

**Figure 1:**
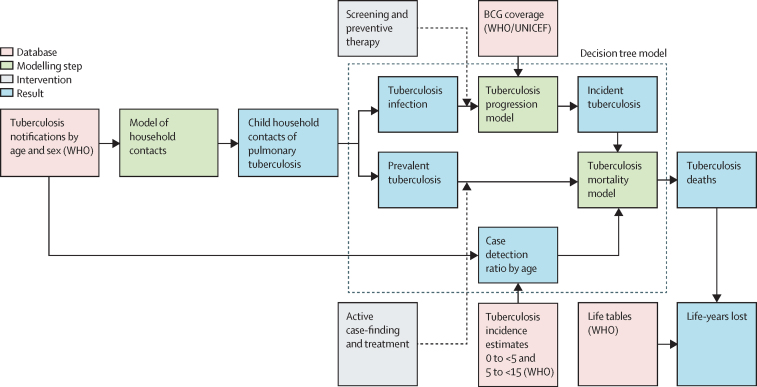
Overview of modelling logic The dotted box shows elements of the overall model that are assessed using the decision tree model. BCG=Bacillus Calmette-Guérin vaccination.

We did a mathematical modelling study in the global population of children younger than 15 years cohabiting with a patient diagnosed with tuberculosis. We assessed the effect of full versus zero HCM coverage, estimating outcomes for demands on the health-care system and child morbidity and mortality. We used a two-stage process, developing regression-based estimates of numbers of child household contacts, and then combining these with a decision tree model to estimate their outcomes (figure 1).

### Interventions and outcomes

We considered three scenarios: scenario A approximates the status quo in which HCM is not routinely done in high-burden settings.^10^, ^11^ In scenario A, we assumed that co-prevalent and incident tuberculosis in children would be detected and treated with the country-specific case detection rate for children cohabiting with patients diagnosed with tuberculosis. Scenario B reflects the long-standing WHO recommendations for the management of tuberculosis households in high-burden settings before 2018.^6^ In this scenario, we assumed that tuberculosis screening for all household contacts younger than 15 years and preventive therapy for all children younger than 5 years and all HIV-positive children younger than 15 years were done. Scenario C extends scenario B to reflect updated recommendations from 2018^7^ by expanding the use of preventive therapy to TST-positive children aged 5–14 years. In scenarios B and C, we do not account for screening or preventive therapy for cohabiting adults, and assume that the case detection rate for incident childhood tuberculosis disease is unaffected by screening activities. To assess maximal impact, we assumed complete intervention coverage, recognising that this is an idealised scenario. We based the yield of co-prevalent tuberculosis in child household contacts on empirical data derived from household contact studies.^5^ In each of these studies, the diagnosis of childhood tuberculosis was made using the diagnostic tools that were available in their setting, with associated imperfect sensitivity and specificity.

As measures of demands on the health-care system, we calculated the number of households, the number of cohabiting children who would be screened, the number who would be given anti-tuberculosis treatment for active disease (including those found through passive case detection), and the number who would receive preventive therapy.

As measures of morbidity and mortality, we calculated the numbers of children who had prevalent disease at the time of the index patient's diagnosis (co-prevalent), who develop incident tuberculosis disease within 1 year (incident), and who die from tuberculosis within 1 year. Our estimates of risk of co-prevalent tuberculosis disease and mortality if treated or untreated were derived from empirical data,^3^, ^5^ reflecting the realities of diagnosing tuberculosis disease in children with imperfect sensitivity and specificity. We also estimated the total years of expected life in cohabiting children to be able to establish years of expected life gained via intervention.

To describe the impact of each intervention scenario, we calculated differences in outcomes of interest between scenarios A and B and between scenarios A and C. Additionally, we calculated the number of household visits, screened children, preventive therapy, and anti-tuberculosis treatment courses per tuberculosis death averted and per case averted. We calculated each outcome for each country under each scenario. Uncertainty was modelled in all input parameters and summaries from 1000 sampled parameter sets reported stratified by age and region as well as globally.

### Model for numbers of contacts

To predict the number of children younger than 5 years and those aged 5–14 years cohabiting with patients diagnosed with tuberculosis of a given age and sex, we developed a Bayesian multivariate regression model based on data from Demographic and Health Surveys from 69 countries with standardised comparable data on household composition. The response data were (simultaneously) the mean number of children in each of the two age groups living with men and women in the age groups of 15–24 years, 25–34 years, 35–44 years, 45–54 years, 55–64 years, and 65 years and older. A sensitivity analysis for India based on a Demographic and Health Survey that included a question on self-reported tuberculosis explored the potential for households of patients diagnosed with tuberculosis to systematically differ, controlling for index patient age and sex. Survey design was accounted for in calculating confidence intervals for contact numbers, and a measurement model included this sample uncertainty in the regression analysis. World Bank data on per capita gross domestic product, life expectancy at birth, infant mortality, population fraction younger than 15 years, population fraction living in urban areas, population density, and total fertility were used as country-level predictors. We ran this model on the most recent World Bank data to predict the number of child household contacts in each country and age group for each of 180 countries, and combined this with WHO age-stratified and sex-stratified tuberculosis notification data for 217 countries and territories.^12^ Missing data were assigned WHO regional averages. Household tuberculosis status was not a major additional influence on numbers of cohabiting children (appendix).

### Decision tree model for child contacts

**Figure 2:**
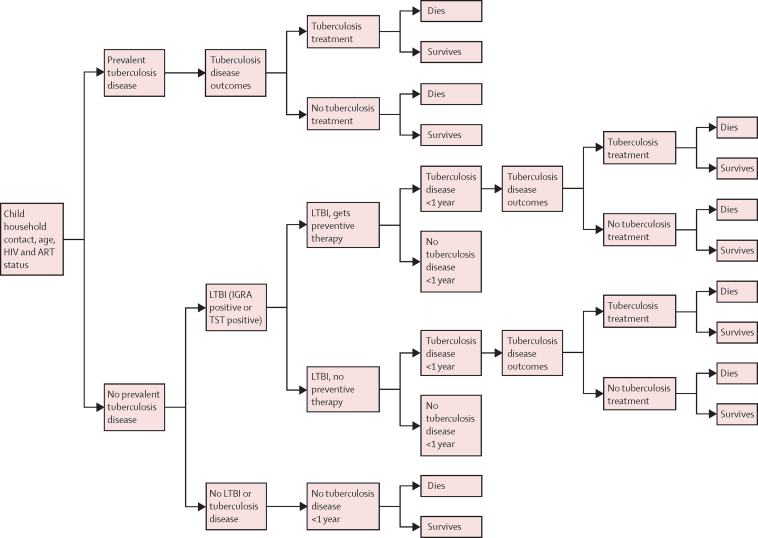
Decision tree model for tuberculosis household contacts ART=antiretroviral therapy. LTBI=latent tuberculosis infection. IGRA=interferon gamma release assay. TST=tuberculin skin test.

A decision tree model was developed, which was based on published models of tuberculosis incidence^13^, ^14^ and mortality^2^ in children, depending on age, HIV and antiretroviral therapy (ART) status, BCG vaccination status, and whether anti-tuberculosis treatment was received (figure 2). These models were extended using systematic review data on tuberculosis disease and latent tuberculosis infection co-prevalence in household tuberculosis contacts by age group and country income,^5^ the efficacy of preventive therapy in preventing tuberculosis disease in children,^15^, ^16^ and life expectancy by age and country (appendix). We assumed no further transmission occurred after HCM.

Data on progression risks were mainly from children judged to have latent tuberculosis infection by TST;^17^ the predicted number latently infected (co-prevalent active tuberculosis excluded) was therefore combined with progression rates to establish incidence. Consistent with these data, preventive therapy was assumed effective only in TST-positive children, with an efficacy based on the variance-weighted average of preventive therapy studies in only TST-positive children identified by systematic review,^15^ and a separate efficacy for children living with HIV.^16^

The probability of children being diagnosed with tuberculosis disease and receiving anti-tuberculosis treatment in the absence of HCM was based on the case detection rate for each age group and country from WHO estimates and notification data.^12^ Because children with tuberculosis cohabiting with patients who have been diagnosed with tuberculosis are probably more likely to receive anti-tuberculosis treatment than average population-matched children with tuberculosis, we used a distribution that interpolated between the population average and an upper-bound case detection rate by an uncertain amount (appendix). We modelled the higher HIV prevalence in children living with patients with tuberculosis who are HIV positive using data on HIV prevalence by age in child household contacts of people with tuberculosis who are HIV positive from Uganda,^18^ and the WHO data on the prevalence of HIV and ART among patients diagnosed with tuberculosis.^12^ We assumed the same ART coverage in child household contacts as in HIV-positive patients with diagnosed tuberculosis (appendix). All analyses were done using the R environment for statistical computing, version 3.4.4.

### Role of the funding source

The funders of the study had no role in the study design, data collection, data analysis, data interpretation, or writing of the report. The corresponding author had full access to all the data in the study and had final responsibility for the decision to submit for publication.

## Results

If HCM was conducted on all patients diagnosed with pulmonary tuberculosis in 2016, the number of households visited globally would have been 5 100 000. This number would have reached an estimated 8 258 000 (75% uncertainty interval [UI] 8 154 000–8 349 000) children younger than 15 years for screening, consisting of 2 789 000 (75% UI 2 744 000–2 834 000) children younger than 5 years and 5 469 000 (75% UI 5 371 000–5 555 000) children aged 5–14 years (table 1). Without HCM (scenario A), we estimated that the children (all those younger than 15 years) would receive 594 200 (75% UI 530 000–649 000) anti-tuberculosis treatment courses, which would increase in scenario B to 862 500 (75% UI 797 600–917 900) and in scenario C to 797 200 (75% UI 734 500–851 100), because some cases in scenario C would be averted by expanded preventive therapy use. Anti-tuberculosis treatment courses resulting from passive detection of incident and co-prevalent cases and anti-tuberculosis treatment courses resulting from HCM identification and treatment of co-prevalent child patients are included. We estimated that 2 543 000 (75% UI 2 497 000–2 588 000) preventive therapy courses would be needed for scenario B and 5 174 000 (75% UI 5 076 000–5 261 000) courses for scenario C.

**Table 1:** Demands on the health-care system for tuberculosis household contact management interventions in children younger than 15 years

	**Scenario A: no household contact management**	**Scenario B: preventive therapy to children younger than 5 years and children who are HIV positive**	**Scenario C: preventive therapy to children younger than 5 years and children who are HIV positive or TST positive**	**Difference between B and A**	**Difference between C and A**
**All children younger than 15 years**					
Children screened	0	8 258 000 (8 154 000–8 349 000)	..	8 258 000 (8 154 000–8 349 000)	..
Anti-tuberculosis treatments	594 200 (530 000–649 000)	862 500 (797 600–917 900)	797 200 (734 500–851 100)	268 300 (235 100–297 300)	202 900 (166 000–236 400)
Preventive therapy courses	0	2 543 000 (2 497 000–2 588 000)	5 174 000 (5 076 000–5 261 000)	2 543 000 (2 497 000–2 588 000)	5 174 000 (5 076 000–5 261 000)
**Children younger than 5 years**					
Children screened	0	2 789 000 (2 744 000–2 834 000)	..	2 789 000 (2 744 000–2 834 000)	..
Anti-tuberculosis treatments	163 200 (151 200–175 300)	295 400 (272 500–315 300)	..	132 200 (115 000–147 800)	..
Preventive therapy courses	0	2 511 000 (2 465 000–2 556 000)	..	2 511 000 (2 465 000–2 556 000)	..
**Children aged 5–14 years**					
Children screened	0	5 469 000 (5 371 000–5 555 000)	..	5 469 000 (5 371 000–5 555 000)	..
Anti-tuberculosis treatments	431 100 (367 900–480 300)	567 100 (506 400–616 900)	501 800 (442 500–552 200)	136 000 (107 700–158 700)	70 690 (38 620–95 280)
Preventive therapy courses	0	31 900 (31 100–32 690)	2 663 000 (2 585 000–2 745 000)	31 900 (31 100–32 690)	2 663 000 (2 585 000–2 745 000)

Data are numbers of children (75% uncertainty interval). Anti-tuberculosis treatments include those resulting from routine services. TST=tuberculin skin test. ..indicates cells for which the number is the same for scenarios B and C—ie, where no additional children were eligible for preventive therapy in scenario C than scenario B.

In scenario A, we estimated 996 500 (75% UI 930 300–1 049 000) co-prevalent and incident tuberculosis cases in children younger than 15 years living with patients with diagnosed tuberculosis, resulting in 133 500 (75% UI 123 400–142 400) deaths (table 2). We estimated that the majority of these tuberculosis cases would be in children aged 5–14 years (613 700 [75% UI 553 000–666 900), whereas the majority of deaths would be in children younger than 5 years (101 000 [92 500–108 200]). Scenario B averted 66 700 (75% UI 59 790–72 370) cases of tuberculosis disease, and scenario C averted 159 500 (75% UI 147 000–170 900) cases. Scenario B averted 103 600 (75% UI 94 480–111 900) deaths, and scenario C averted 108 400 (75% UI 98 800–116 700) deaths; in both scenarios B and C, most of these averted deaths were in children younger than 5 years. Scenario B gained 7 006 000 (75% UI 6 373 000–7 567 000) life-years, and scenario C gained 7 305 000 (75% UI 6 663 000–7 874 000) life-years; in both scenarios, the most life-years were gained in children younger than 5 years.

**Table 2:** Morbidity and mortality outcomes for tuberculosis household contact management interventions in children younger than 15 years

	**Scenario A: no household contact management**	**Scenario B: preventive therapy to children younger than 5 years and children who are HIV positive**	**Scenario C: preventive therapy to children younger than 5 years and children who are HIV positive or TST positive**	**Difference between B and A**	**Difference between C and A**
**All children younger than 15 years**					
Tuberculosis cases	996 500 (930 300 to 1 049 000)	929 800 (863 900 to 983 300)	837 000 (771 700 to 892 400)	−66 700 (−72 370 to −59 790)	−159 500 (−170 900 to −147 000)
Tuberculosis deaths	133 500 (123 400 to 142 400)	29 840 (27 300 to 31 750)	25 130 (22 850 to 26 880)	−103 600 (−111 900 to −94 480)	−108 400 (−116 700 to −98 800)
Total life expectancy (years)	526 200 000 (519 400 000 to 532 200 000)	533 200 000 (526 400 000 to 539 300 000)	533 500 000 (526 700 000 to 539 600 000)	7 006 000 (6 373 000 to 7 567 000)	7 305 000 (6 663 000 to 7 874 000)
**Children younger than 5 years**					
Tuberculosis cases	382 800 (358 100 to 404 300)	318 000 (294 500 to 338 800)	..	−64 800 (−70 490 to −57 820)	..
Tuberculosis deaths	101 000 (92 500 to 108 200)	17 550 (15 520 to 19 000)	..	−83 460 (−90 150 to −75 800)	..
Total life expectancy (years)	182 900 000 (179 800 000 to 185 900 000)	188 600 000 (185 400 000 to 191 700 000)	..	5 724 000 (5 181 000 to 6 188 000)	..
**Children aged 5–14 years**					
Tuberculosis cases	613 700 (553 000 to 666 900)	611 800 (551 100 to 664 900)	519 000 (459 300 to 569 700)	−1906 (−2037 to −1765)	−94 710 (−103 700 to −84 700)
Tuberculosis deaths	32 480 (27 460 to 36 430)	12 290 (10 810 to 13 440)	7584 (6611 to 8334)	−20 180 (−23 180 to −16 090)	−24 900 (−28 450 to −20 320)
Total life expectancy (years)	343 400 000 (337 000 000 to 348 800 000)	344 700 000 (338 300 000 to 350 300 000)	344 900 000 (338 600 000 to 350 600 000)	1 282 000 (1 016 000 to 1 479 000)	1 581 000 (1 281 000 to 1 816 000)

Data are number (75% uncertainty interval). Tuberculosis cases, deaths, and life expectancy all include contributions from incident and co-prevalent cases of tuberculosis. .. indicates cells for which the number is the same for scenarios B and C because no additional children were eligible for preventive therapy in scenario C than scenario B. TST=tuberculin skin test.

The WHO southeast Asia region had the largest share of preventable deaths, followed by the African region, the western Pacific region, the eastern Mediterranean region, and the region of the Americas and European region (appendix).

Globally, only 3% of tuberculosis deaths in children cohabiting with patients with diagnosed tuberculosis with no HCM were estimated to be HIV positive, but 7% were estimated in the WHO African region. In scenario A, more than 70% of tuberculosis cases in children were co-prevalent upon index case notification, rather than incident during the subsequent year, with a similar split in anti-tuberculosis treatment courses given to co-prevalent versus incident child tuberculosis cases (appendix). The increases in anti-tuberculosis treatment courses for co-prevalent cases found by HCM (scenarios B and C) were partially offset by anti-tuberculosis treatment courses averted through preventive-therapy-mediated reductions in incidence (appendix).

**Figure 3:**
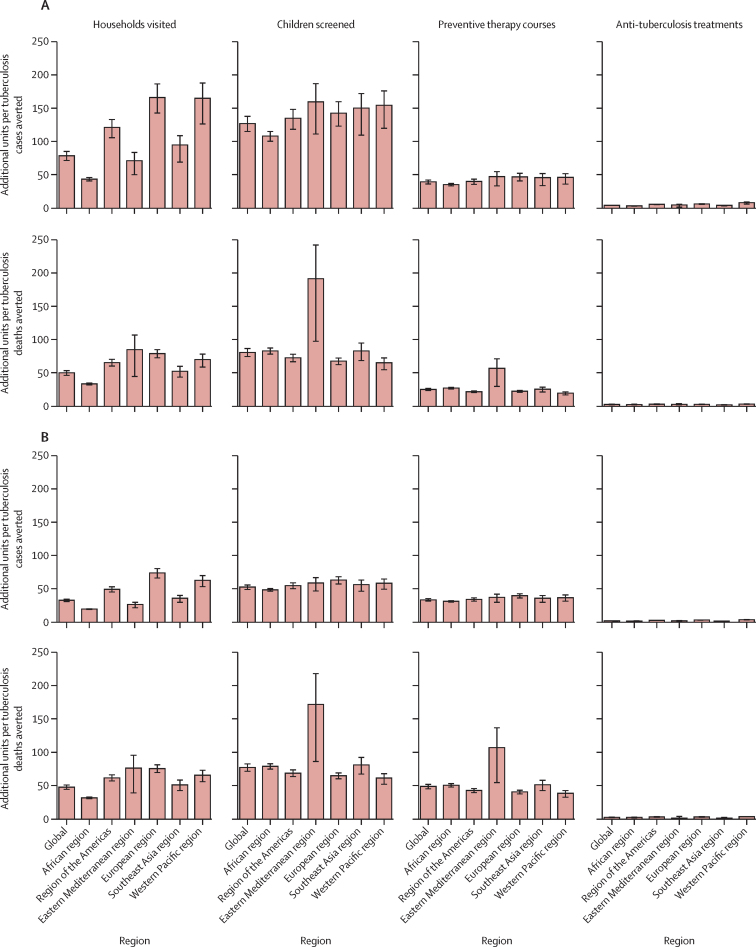
Impact of fully implemented household contact management in children younger than 15 years as incremental demands on the health-care system required to avert one tuberculosis case or death (A) Scenario B versus scenario A. (B) Scenario C versus scenario A. Error bars show 75% uncertainty interval. Scenario A=no household contact management. Scenario B=preventive therapy to children younger than 5 years and children who are HIV positive. Scenario C=preventive therapy to children younger than 5 years and children who are HIV positive or TST positive.

In scenario B versus scenario A, globally, for every child tuberculosis case averted, 78 (75% UI 70–85) households were visited, 126 (75% UI 113–138) children were screened, 42 (75% UI 35–39) preventive therapy courses were given, and an additional 4 (75% UI 3–5) anti-tuberculosis treatment courses were given (figure 3). In scenario C versus scenario A, globally, for every child tuberculosis case averted, 32 (75% UI 30–35) households were visited, 52 (75% UI 48–56) children were screened, 33 (75% UI 30–35) preventive therapy courses were given, and 1 (75% UI 1–2) additional anti-tuberculosis treatment course was given.

In scenario B versus scenario A, globally, for every child tuberculosis death averted, 49 (75% UI 46–54) households were visited, 81 (75% UI 74–87) children were screened, 25 (75% UI 23–27) preventive therapy courses were given, and an additional 3 (75% UI 2–3) anti-tuberculosis treatment courses were given (figure 3). In scenario C versus scenario A, globally, for every child tuberculosis death averted, 48 (75% UI 44–52) households were visited, 77 (75% UI 71–83) children were screened, 48 (75% UI 44–52) preventive therapy courses were given, and an additional 2 (75% UI 2–2) anti-tuberculosis treatment courses were given (figure 3).

## Discussion

In this mathematical modelling study, we found that HCM implemented at full scale might prevent about 110 000 deaths and about 160 000 cases of tuberculosis disease in children every year. The deaths averted would amount to more than 7 million expected life-years saved. Children younger than 5 years would derive the greatest benefit, comprising two in every five averted cases and three in every four of the averted deaths. Extending use of preventive therapy to TST-positive children older than 5 years, consistent with 2018 updated WHO guidelines,^7^ would more than double the number of paediatric cases averted.

We have considered the scenario in which long-time recommended interventions are implemented for children exposed at home to tuberculosis, with perfect coverage of both screening and treatments. We have not considered the best approaches for achieving these ends, and varying approaches would probably be needed in different contexts. We have also not considered the true economic costs of these activities, nor considered the quality-of-life gains from reductions in tuberculosis disease and its sequelae. Our aim was to project the expected benefits, in terms of the reduction of tuberculosis disease and death in children, that could be achieved through child-specific household management interventions. Certainly, the numbers of children screened and given preventive therapy estimated by this model are large, and the numbers of children screened and treated are far greater than those that most tuberculosis programmes achieve, but some examples of successful implementations in resource-limited settings do exist.^19^ Quantifying this additional workload is a first step for global planning: projecting the action required—ie, the numbers of household visits, children screened, and preventive therapy courses needed. The numbers of children screened that was required to avert one tuberculosis death compare favourably with estimates of numbers needed to screen to find one case for other tuberculosis screening activities.^20^

These results can be used to help identify the envelope within which alternative service provisions might be deemed cost-effective in different countries. Evidence shows that interventions generating health gain at less than US$200 per disability-adjusted life-year (DALY) averted would be deemed cost-effective in many countries,^21^ even those classified as amongst the poorest in the world. Assuming that each death avoided generates 30 DALYs averted per child saved after discounting, a country programme able to pay $200 per DALY averted should be willing to spend up to $6000 ($200 times 30) to save one child's life. The question is whether the costs required to do this (here estimated for scenario B as making 50 household visits, screening 80 children for active tuberculosis, administering 25 preventive therapy courses, and giving three courses of anti-tuberculosis treatment) plus any net downstream costs of treatments can be delivered within this budget. Analyses of the health system costs of contact screening and diagnosing and treating tuberculosis infection from Uganda, Malaysia, and Vietnam suggest that this is a highly feasible target.^22^, ^23^, ^24^ If in a particular setting the costs of intervention exceed this amount, then a priority task becomes how to deliver the necessary interventions more efficiently.

To reliably establish the cost-effectiveness of scaling up household contact management across different countries, full costing of those interventions would be required, as well as establishing measures of morbidity and mortality such as DALYs. One would need to consider the feasibility of delivery given constraints within health-care systems and uptake by different population groups. All these factors are context specific and could depend importantly upon the scale of delivery (eg, average costs might fall initially as intervention programmes are scaled up, but rise as they are taken into hard-to-reach areas). This scaling up of delivery should be the focus for further research, in which epidemiological and health economic modellers work hand-in-hand with implementation scientists. Novel models of service provision might have advantages, such as the use of community health workers or integration with maternal and child health programmes; these will need to be assessed using implementation research. Mandatory monitoring and reporting are often necessary to drive implementations of this kind. The health technology landscape is also changing rapidly. Shorter, more patient-acceptable and more implementable treatments for tuberculosis infection would help to increase uptake of preventive therapy and promote adherence to treatment completion.^25^ Additionally, the development of tests that could better identify those at increased risk of progressing to tuberculosis disease would mean that fewer contacts would require preventive therapy.^26^

We have considered full coverage of the intervention scenarios, but we used yield data from household screening activities that used imperfectly sensitive and specific approaches to diagnosing tuberculosis disease in child household contacts, after realistic delays in reaching households (during which tuberculosis could have developed or deaths occurred). These imperfect approaches are also the basis of entry to the studies on which we base our estimates of risk of death with treated and untreated tuberculosis disease. We have modelled TST-positivity rather than true latent tuberculosis infection because this is what most of the data on household infection rates and progression rates are based on. We might therefore have been conservative in our assessment of preventive therapy effect, in assuming no benefit to TST-negative children.

Our analysis is also subject to several simplifications. Our scope explicitly excluded benefits and treatments in cohabiting adults, who would normally be screened for tuberculosis disease. The treatment of these adults is anticipated to increase effects with limited increases in demand on the health-care system. We also limited HCM to patients with pulmonary tuberculosis; HCM for other forms of tuberculosis might provide benefits by identifying children at risk from a common exposure. We assumed that the number of cohabiting children depended on adult age and sex but was not different for households of patients with diagnosed tuberculosis. An analysis for India (appendix) suggested this assumption to be reasonable. We also made the simplifying assumptions that all diagnosed patients with tuberculosis lived in households and that none shared a household, which could potentially have overestimated the number of children living in households affected by tuberculosis. However, the proportion of diagnosed adults sharing a house is small,^5^ and by only considering children cohabiting with patients with tuberculosis, we conservatively underestimate the reach of contact management, which should include non-cohabiting young children who have spent significant time in the household. This time spent might include contact with caregivers, such as grandparents, who do not live with the child. We were similarly conservative in assuming that household management would not improve household awareness of tuberculosis and therefore improve case detection in subsequent incident disease in children. We also did not make allowance for any reductions in life expectancy in children living with HIV (although children with HIV contributed a small fraction of deaths). Furthermore, we did not consider multidrug-resistant tuberculosis, which is expected to affect about 3% of children with tuberculosis;^27^ children with multidrug-resistant tuberculosis comprise a group with different case detection rates and treatment and preventive therapy outcomes. Given how few children with drug-resistant tuberculosis are diagnosed and treated, the effect of appropriate household management might be even more pronounced than for patients with drug-susceptible tuberculosis. A study published in 2018 estimated that with universal HCM after the diagnosis of an adult with multidrug-resistant tuberculosis, 12 times as many children would be treated for multidrug-resistant tuberculosis than are currently.^27^

Our analysis is the first to project the total global health impact that could be achieved by child-targeted household management. We emphasise uncertainty propagation, and a detailed model of tuberculosis natural history in children. Our study brings together estimates of household structure and a model of intervention effect and outcomes focused on children, and it uses evidence from systematic reviews from the past 5 years.^3^, ^5^, ^15^, ^16^ Yuen and colleagues^28^ estimated numbers of children with co-prevalent disease in household contacts, obtaining similar results to ours. A cost-effectiveness modelling study^29^ considered tuberculosis infection testing and preventive therapy to reduce tuberculosis incidence in cohorts of child household contacts younger than 5 years for a particular setting, concluding that all strategies were cost-effective. We included both these elements—the substantial benefits from household management to co-prevalent paediatric cases and the reductions in incidence from preventive therapy—and extended our analysis to 217 countries and territories. The balance between cases averted through preventive therapy and deaths averted depends strongly on the co-prevalence levels from a systematic review;^5^ lower co-prevalence in some settings would shift the benefits of HCM towards those derived from case prevention rather than case finding. However, our lower projected demands on the health-care system per death averted than per case averted indicates that HCM achieves substantial benefit through its case-finding component and cannot simply be considered as a mode of delivering preventive therapy. Benchmarking our estimates of cases averted against global incidence estimates of about 1 million tuberculosis cases in children per year suggests that 16% of incidence could be avoided by full HCM implementation. Although a direct comparison is not appropriate between the global mortality estimates of 250 000 child tuberculosis deaths per year and our estimate of 110 000 deaths averted after 1 year of full HCM because the case-finding intervention component includes prevalent cases incident in previous years, this finding does indicate the potential for a large impact on mortality.

HCM for tuberculosis has the potential to prevent substantial morbidity and mortality in children, with impact-for-effort that compares favourably with other interventions. The low global coverage of this effective intervention needs to increase; funding and implementation research to enable this should be prioritised.

For the **data handling scripts** see https://github.com/petedodd/PINT

## Data sharing

## References

[1] WHO (2017). Global tuberculosis report 2017. http://www.who.int/tb/publications/global_report/en/.

[2] Dodd PJ, Yuen CM, Sismanidis C, Seddon JA, Jenkins HE (2017). The global burden of tuberculosis mortality in children: a mathematical modelling study. Lancet Glob Health.

[3] Jenkins HE, Yuen CM, Rodriguez CA (2017). Mortality in children diagnosed with tuberculosis: a systematic review and meta-analysis. Lancet Infect Dis.

[4] Chiang SS, Khan FA, Milstein MB (2014). Treatment outcomes of childhood tuberculous meningitis: a systematic review and meta-analysis. Lancet Infect Dis.

[5] Fox GJ, Barry SE, Britton WJ, Marks GB (2013). Contact investigation for tuberculosis: a systematic review and meta-analysis. Eur Respir J.

[6] WHO (2012). Recommendations for investigating contacts of persons with infectious tuberculosis in low- and middle-income countries. http://www.who.int/tb/publications/2012/contact_investigation2012/en/.

[7] WHO (2018). Latent TB infection: updated and consolidated guidelines for programmatic management. http://www.who.int/tb/publications/2018/latent-tuberculosis-infection/en/.

[8] Jagger A, Reiter-karam S, Hamada Y, Getahun H (2018). National policies on the management of latent tuberculosis infection: review of 98 countries. Bull World Health Organ.

[9] Rodriguez CA, Sasse S, Yuengling KA, Azzawi S, Becerra MC, Yuen CM (2017). A systematic review of national policies for the management of persons exposed to tuberculosis. Int J Tuberc Lung Dis.

[10] Szkwarko D, Hirsch-Moverman Y, Du Plessis L, Du Preez K, Carr C, Mandalakas AM (2017). Child contact management in high tuberculosis burden countries: a mixed-methods systematic review. PLoS One.

[11] Hill PC, Rutherford ME, Audas R, van Crevel R, Graham SM (2011). Closing the policy-practice gap in the management of child contacts of tuberculosis cases in developing countries. PLoS Med.

[12] WHO (2018). Tuberculosis. http://www.who.int/tb/country/data/download/en/.

[13] Dodd PJ, Gardiner E, Coghlan R, Seddon JA (2014). Burden of childhood tuberculosis in 22 high-burden countries: a mathematical modelling study. Lancet Glob Health.

[14] Dodd PJ, Sismanidis C, Seddon JA (2016). Global burden of drug-resistant tuberculosis in children: a mathematical modelling study. Lancet Infect Dis.

[15] Ayieko J, Abuogi L, Simchowitz B, Bukusi EA, Smith AH, Reingold A (2014). Efficacy of isoniazid prophylactic therapy in prevention of tuberculosis in children: a meta-analysis. BMC Infect Dis.

[16] Zunza M, Gray DM, Young T, Cotton M, Zar HJ (2017). Isoniazid for preventing tuberculosis in HIV-infected children. Cochrane Database Syst Rev.

[17] Marais BJ, Gie RP, Schaaf HS (2004). The natural history of childhood intra-thoracic tuberculosis: a critical review of literature from the pre-chemotherapy era. Int J Tuberc Lung Dis.

[18] Martinez L, Shen Y, Handel A (2018). Effectiveness of WHO's pragmatic screening algorithm for child contacts of tuberculosis cases in resource-constrained settings: a prospective cohort study in Uganda. Lancet Respir Med.

[19] Fox GJ, Nhung NV, Sy DN (2018). Household-contact investigation for detection of tuberculosis in Vietnam. N Engl J Med.

[20] Shapiro AE, Chakravorty R, Akande T, Lonnroth K, Golub JE (2013). A systematic review of the number needed to screen to detect a case of active tuberculosis in different risk groups. http://www.who.int/tb/Review3NNS_case_active_TB_riskgroups.pdf.

[21] Woods B, Revill P, Sculpher M, Claxton K (2016). Country-level cost-effectiveness thresholds: initial estimates and the need for further research. Value Health.

[22] Minh HV, Mai VQ, Nhung NV (2017). Costs of providing tuberculosis diagnosis and treatment services in Viet Nam. Int J Tuberc Lung Dis.

[23] Atif M, Sulaiman SAS, Shafie AA, Ali I, Asif M (2012). Tracing contacts of TB patients in Malaysia: costs and practicality. Springerplus.

[24] Sekandi JN, Dobbin K, Oloya J, Okwera A, Whalen CC, Corso PS (2015). Cost-effectiveness analysis of community active case finding and household contact investigation for tuberculosis case detection in urban Africa. PLoS One.

[25] Villarino ME, Scott NA, Weis SE (2015). Treatment for preventing tuberculosis in children and adolescents: a randomized clinical trial of a 3-month, 12-dose regimen of a combination of rifapentine and isoniazid. JAMA Pediatr.

[26] Zak DE, Penn-Nicholson A, Scriba TJ (2016). A blood RNA signature for tuberculosis disease risk: a prospective cohort study. Lancet.

[27] Jenkins HE, Yuen CM (2018). The burden of multidrug-resistant tuberculosis in children. Int J Tuberc Lung Dis.

[28] Yuen CM, Jenkins HE, Chang R, Mpunga J, Becerra MC (2016). Two methods for setting child-focused tuberculosis care targets. Public Health Action.

[29] Mandalakas AM, Hesseling AC, Gie RP, Schaaf HS, Marais BJ, Sinanovic E (2013). Modelling the cost-effectiveness of strategies to prevent tuberculosis in child contacts in a high-burden setting. Thorax.

