# Supplementary material for: Potential effect of household contact management on childhood tuberculosis: a mathematical modelling study
Source: Lancet Glob Health. 2018 Sep 25;6(12):e1329–38. doi: 10.1016/S2214-109X(18)30401-7 (PMC6227381; doi:10.1016/S2214-109X(18)30401-7)
Supplement: Supplementary appendix [file mmc1.pdf]

THE LANCET
Global Health

Supplementary appendix

This appendix formed part of the original submission and has been peer reviewed. We post it as supplied by the authors.

Supplement to: Dodd PJ, Yuen CM, Becerra MC, Revill P, Jenkins HE, Seddon JA. Potential effect of household contact management on childhood tuberculosis: a mathematical modelling study. Lancet Glob Health 2018; published online Sept 25. http://dx.doi.org/10.1016/S2214-109X(18)30401-7.

Appendix - The potential impact of household contact management on childhood tuberculosis: a mathematical modelling study

P.J. Dodd, C.M. Yuen, M.C. Becerra, P. Revill, H.E. Jenkins, J.A. Seddon

Input data and preparation **2**
DHS data 2
Number of children in households 2
Are households of TB patients different? 5
World Bank covariate data 6
Regression analysis of number of children in households **7**
Mathematical modelling **9**
Additional input data 9
Pulmonary vs extrapulmonary disease 9
Decision tree model structure 11
Modelling approach and evidence 12
Number of household contacts 12
Prevalence and incidence in household contacts 13
Case detection 13
HIV 13
Mortality, life expectancy and IPT 14
Model parameters, distributions and evidence 16
Probabilistic sensitivity analysis 19
Results **20**
Regional and global results 20
Supplementary Figure 23
Availability of data and analysis code 23
References **24**

Input data and preparation

DHS data

Data for 69 countries were obtained from the Demographic and Health Surveys (DHS) programme (https://dhsprogram.com).

The countries included were: Albania, Armenia, Azerbaijan, Burundi, Benin, Burkina Faso, Bangladesh, Bolivia (Plurinational State of), Brazil, Central African Republic, Côte d'Ivoire, Cameroon, Democratic Republic of the Congo, Congo, Colombia, Comoros, Dominican Republic, Egypt, Ethiopia, Gabon, Ghana, Guinea, Guatemala, Guyana, Honduras, Haiti, Indonesia, India, Jordan, Kazakhstan, Kenya, Kyrgyzstan, Cambodia, Liberia, Lesotho, Morocco, Republic of Moldova, Madagascar, Maldives, Mali, Mozambique, Malawi, Namibia, Nigeria, Nicaragua, Nepal, Pakistan, Peru, Philippines, Paraguay, Rwanda, Senegal, Sierra Leone, Sao Tome and Principe, Swaziland, Chad, Togo, Tajikistan, Timor-Leste, Turkey, United Republic of Tanzania, Uganda, Ukraine, Uzbekistan, Viet Nam, Yemen, South Africa, Zambia, Zimbabwe.

The counts of these countries by World Health Organisation (WHO) regions were as shown in Table 1.

Table 1: Count of DHS surveys included by WHO region.

AFR	AMR	EMR	EUR	SEA	WPR
34	11	5	10	6	3

For each survey: country, year, household ids, age, sex, cluster ids, strata ids, sample weights were extracted for all records and merged. The merged dataset included records for approximately 4.4 million individuals living in approximately 0.9 million households. Ages were binned into categories: [0,5), [5,15), [15,25), [25,35), [35,45), [45,55), [55,65), [65,Inf). This compiled dataset cannot be made available by us due to its licensing requirements, but the raw data can be freely applied for at https://dhsprogram.com/data/.

Number of children in households

The number of children aged [0,5) and [5,15) in each household was computed. For each survey, the mean number (and variance) of children in each age category cohabiting with individuals of each age category and sex was computed. The survey design was accounted using the R package *survey* and specifying the weights, and cluster and strata ids, as per the DHS manual.

The mean number of cohabiting children in age groups [0,5) and [5,15) for individuals of given age and sex is shown in Figures 1 and 2 respectively.

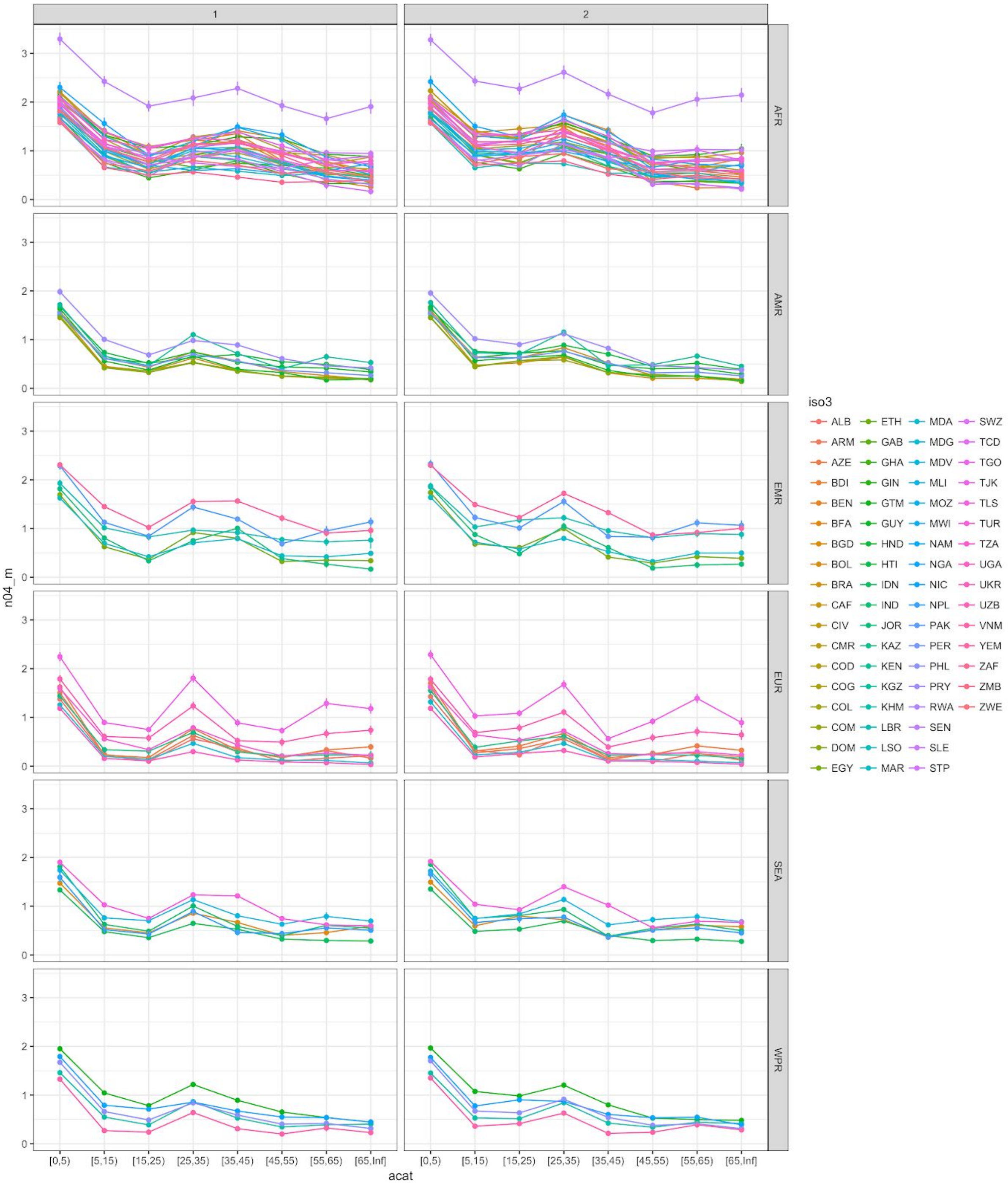

Figure 1: Mean number of children age [0,5) years living with individuals of given age and sex. 1=male, 2=female. Data are grouped by WHO region. Error bars are 95% confidence intervals.

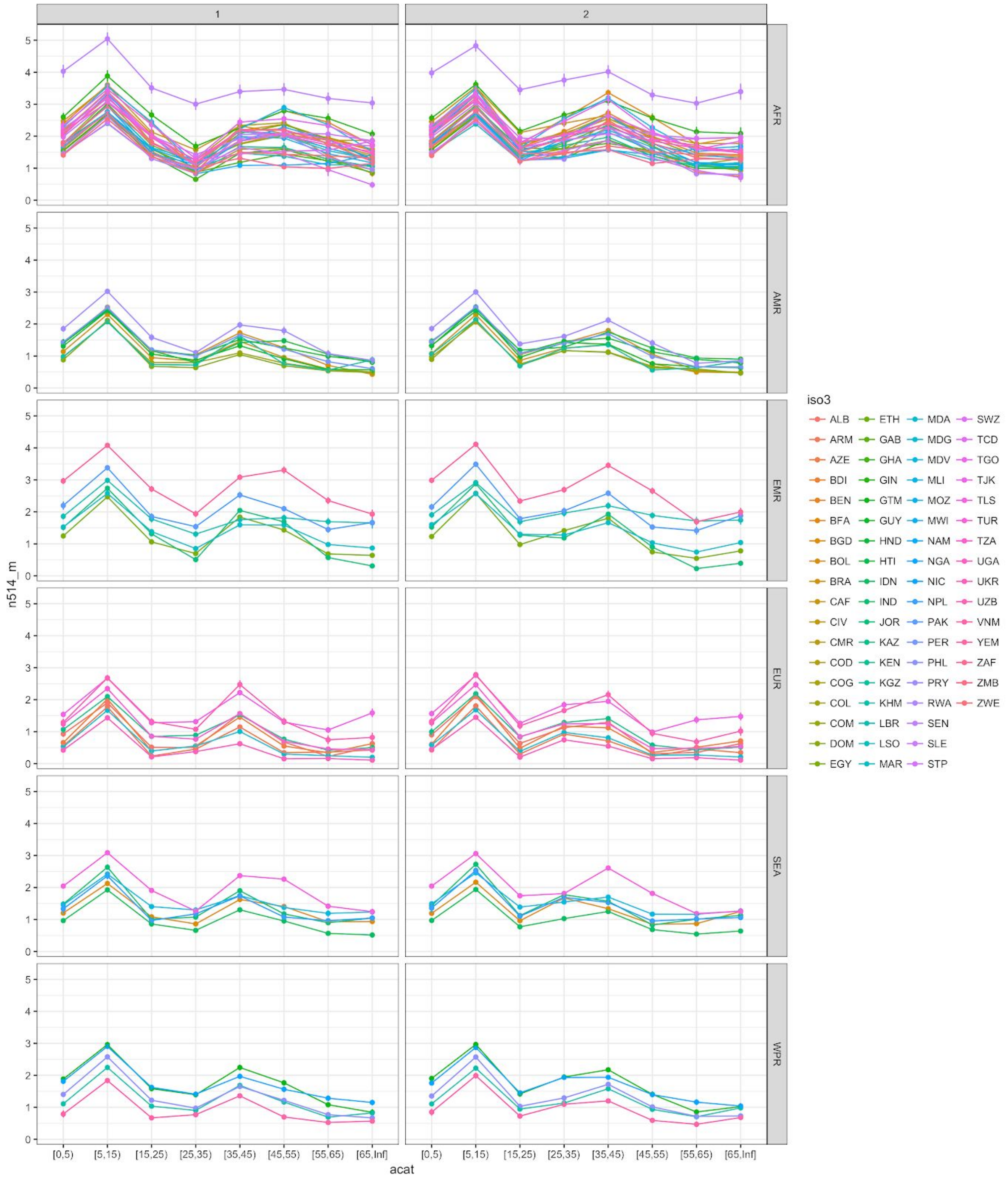

Figure 2: Mean number of children age [5,15) years living with individuals of given age and sex. 1=male, 2=female. Data are grouped by WHO region. Error bars are 95% confidence intervals.

Are households of TB patients different?

We are ultimately interested in predicting the number of children found in households of TB patients of a given age and sex, and it is possible that TB predicts household size and composition independently of age, sex and other variables. Often TB has different epidemiology in rural vs urban populations, so differences by rural/urban classification are also of interest.

Data to explore these patterns generally are limited. However, the large Indian DHS dataset included a self-reported TB prevalence question. We used this to briefly explore whether the household composition of TB patients is systematically different to non-TB patients. We graphed a (generalized additive model [GAM]) smoothed number of cohabiting children (age [0,5) in Figure 3, age [5,15) in Figure 4), together with uncertainty bounds, additionally stratifying by rural/urban classification. There were small differences for children age [5,15), and other settings may be different, but we were generally reassured in proceeding under the assumption that the numbers of cohabiting children cohabiting with TB patients are not substantially different to those cohabiting with non-TB patients of a given age and sex.

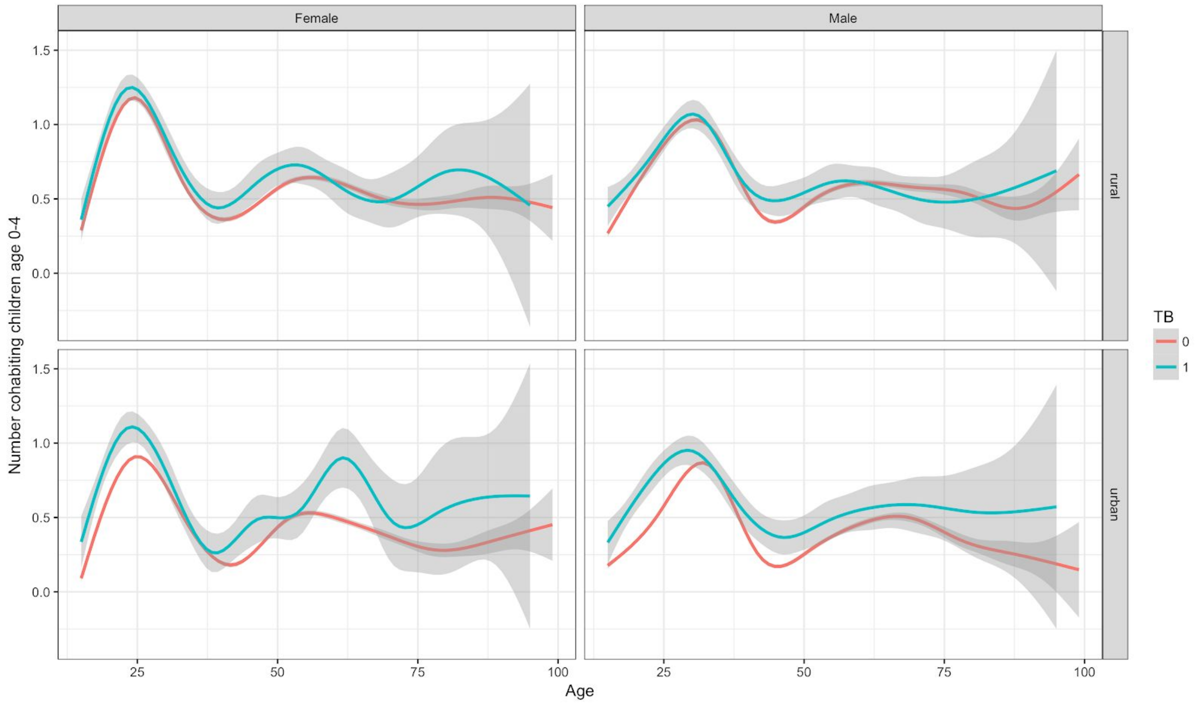

Figure 3: Smoothed number of cohabiting children age [0,5) by age (over 15), sex, rural/urban classification and self-reported TB status for the Indian DHS survey.

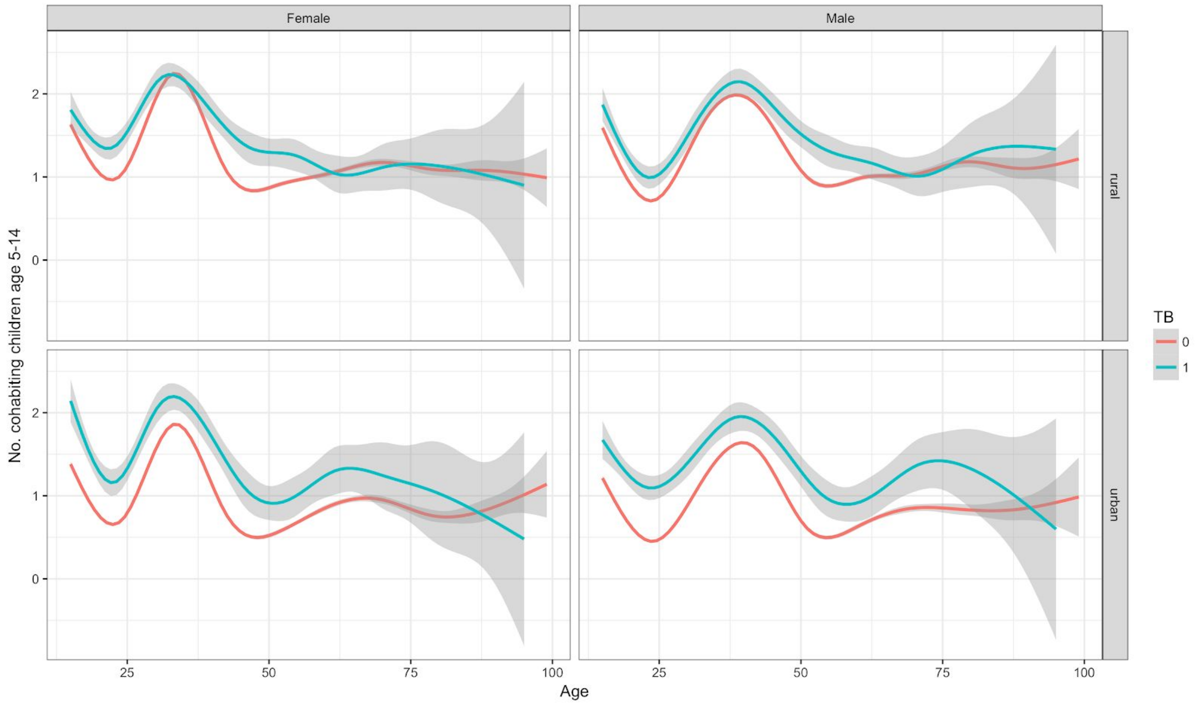

Figure 4: Smoothed number of cohabiting children age [5,15) by age (over 15), sex, rural/urban classification and self-reported TB status for the Indian DHS survey.

World Bank covariate data

Using the R package *wbstats* we gathered (11/01/2018) World Bank data on indicators plausibly related to household structure that were complete across all relevant years (i.e. DHS survey years for each country), namely: per capita GDP, life-expectancy at birth, infant mortality, total population, population under 15 years of age, percentage of population living in urban areas, population density, and total fertility (i.e. indicators: NY.GDP.PCAP.KD, SP.DYN.LE00.IN, SP.DYN.IMRT.IN, SP.POP.TOTL, SP.POP.0014.TO, SP.URB.TOTL.IN.ZS, EN.POP.DNST, SP.DYN.TFRT.IN). The total population and population under 15 years of age were only used to compute the proportion of the population under 15 years of age. We supplemented these quantities with a categorical variable for the WHO region.

One covariate dataset was prepared for the countries with DHS surveys, matching the World Bank year to the DHS survey year; another covariate dataset was prepared for prediction using the most recent year for which data was available for each available country.

Regression analysis of number of children in households

We used multivariate linear regression to model the log-transformed expected number of children aged [0,5) and [5,15) that individuals of given age and sex share households with in each country. To account for the uncertainty arising from sampling error in the DHS survey, we used a Bayesian hierarchical approach with a Normal measurement error. The underlying pattern of means by index case age and sex (e.g. as in Figure 1) was flexibly modelled as multivariate normal (in log space), and the country-level covariates described in the previous section were used.

More formally, we used a model:

$z_i \sim N(y_i, \varepsilon_i^2)$	(measurement model for sampling error)
$y_i \sim MVN(X_i\beta, \Sigma)$	(multivariate normal regression for log-means)
$\beta_{ij} \sim N(0, B_{ij}^2)$	(prior for regression coefficients)
$\Sigma \sim IW(\nu, \Psi)$	(prior for covariance: inverse-Wishart)

Here: i is and index ranging over countries; z_i are the vectors (length = No. ages $\times$ 2 sexes) of observed log-means in the DHS data with ε_i^2 the corresponding vector sample variances for each survey; y_i are the underlying mean vectors for each country, modelled as depending on the matrices X_i of covariates for country i, and with variance-covariance Σ. Normal priors around 0 were assumed for the regression coefficients, with variance 25. An inverse-Wishart distribution with $\nu = 5$ and $\Psi = 1/20$ was used as prior for the covariance matrix Σ. A Gibbs sampling scheme implemented in the R package *mvregerr* (https://github.com/petedodd/mvregerr) was employed to generate 1000 samples (individual parameter chains suggested this was sufficient for convergence). Prediction samples representing both parameter and prediction uncertainty were simultaneously gathered for countries without DHS surveys and using the most recent World Bank data for updated predictions in countries with DHS surveys. This prediction dataset was reduced to predictions (and their uncertainty) for the mean number of cohabiting children in ages [0,5) and [5,15) years a person of a given age and sex and country.

The model fit for children [0,5) years can be seen in Figure 5. The prediction errors from applying prediction to the years and countries of the DHS surveys for children [0,5) years can be seen in Figure 6. The counties with worst predictions are outliers to the general pattern (labelled in Figure 7). Fits for children age [5,15) years were comparable.

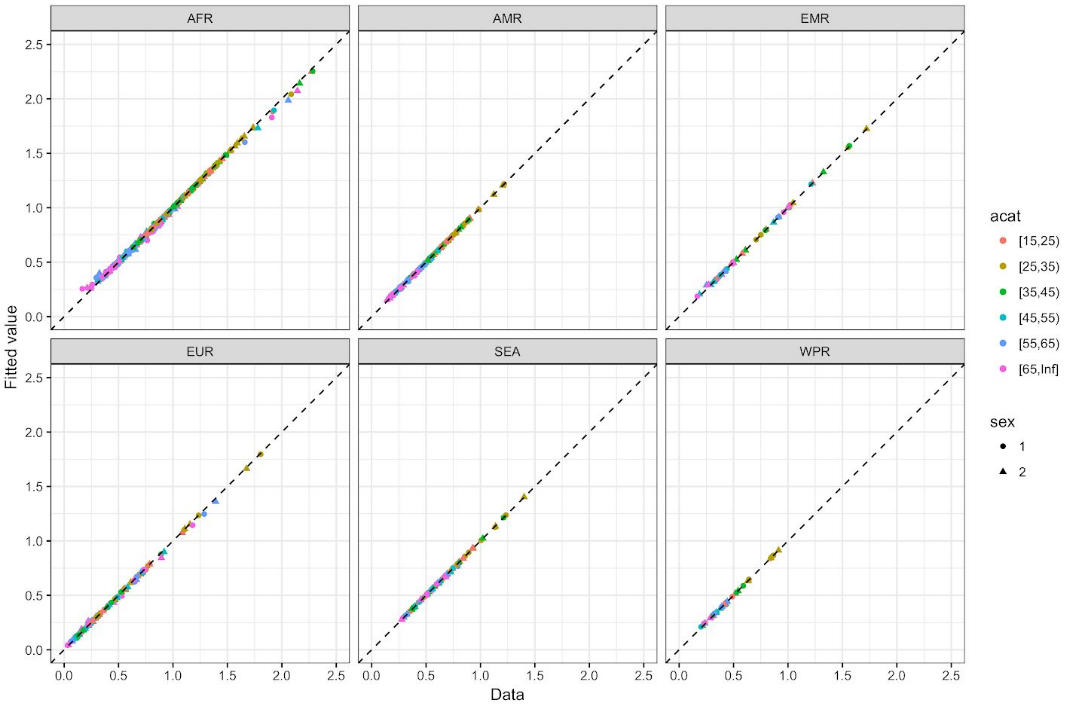

Figure 5: Regression model fit on DHS data for children age [0,5) years.

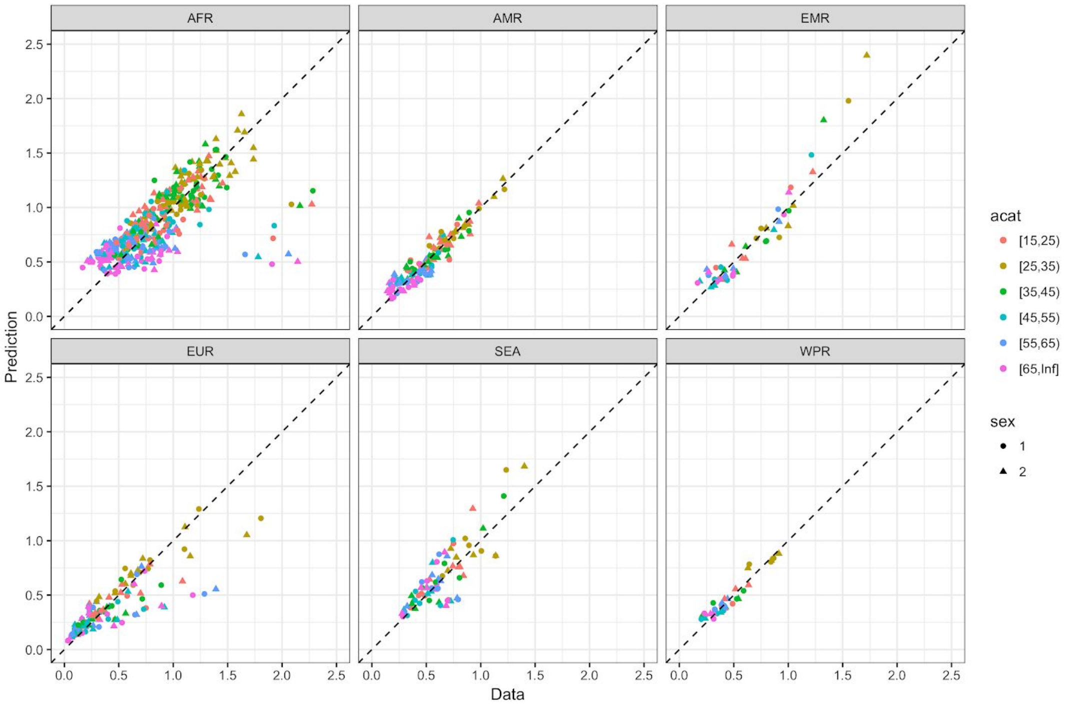

Figure 6: Regression model predictions on DHS data for children age [0,5) years.

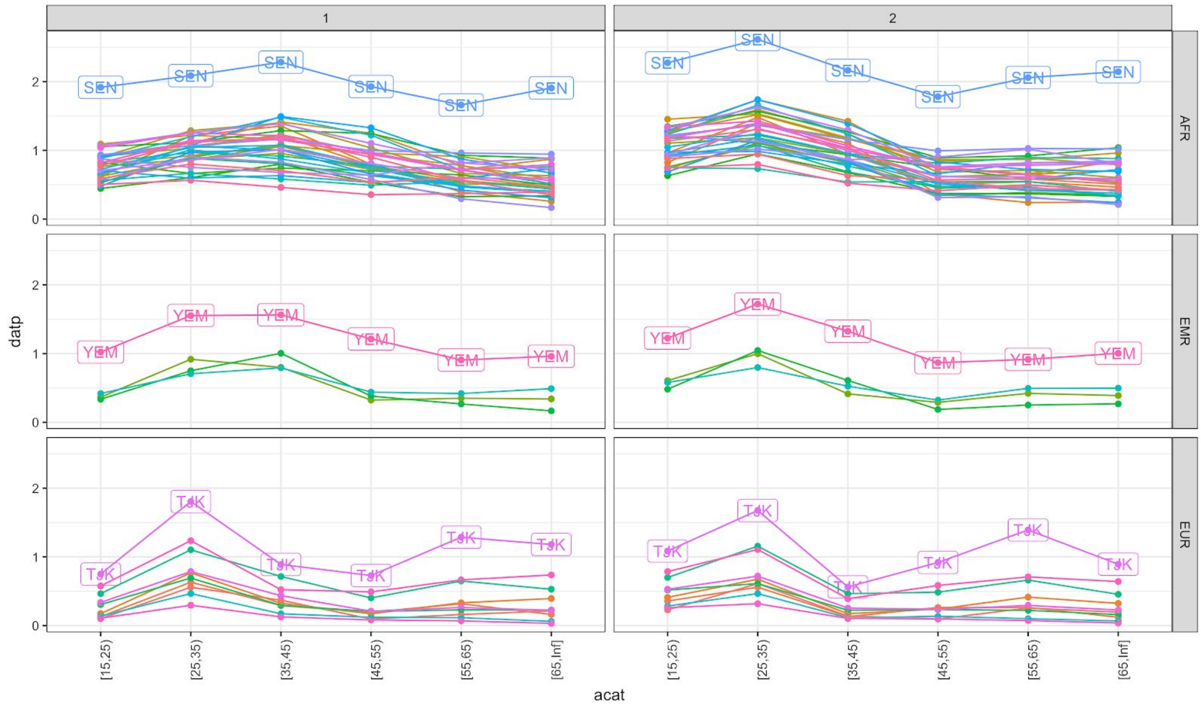

Figure 7: Original DHS data: countries with poor model predictions for children age [0,5) years are labelled.

Mathematical modelling

Additional input data

We obtained the WHO TB notification and burden estimate data available from http://www.who.int/tb/country/data/download/en/ (downloaded 11/01/2018). We also obtained the WHO/UNICEF Joint Report Form data on BCG coverage by country and year from http://www.who.int/immunization/monitoring_surveillance/data/en/ (downloaded 11/01/2018). Missing data were filled with coverages from the nearest year. BCG vaccination coverage by year was converted into BCG coverage in children at each at the present. World Bank country income classifications were from http://databank.worldbank.org/data/download/site-content/CLASS.xls (downloaded 15/03/2018).

The WHO Global Health Observatory database was interrogated (11/01/2018) using the R package *rgho*, to obtain the obtain the life expectancy for all available countries in 2015 for age groups: [0,1), [1,5), [5,10), [10,15) years. The mean life expectancy was taken over both sexes.

Pulmonary vs extrapulmonary disease

WHO notification data up to and including 2012 included age- and sex stratified data on whether new notifications were smear-positive, smear-negative or extrapulmonary. We aggregated adult data over all complete years by country, classifying either pulmonary or extrapulmonary (see Figure 8), and restricted to countries with more than 200 cases included in their data.

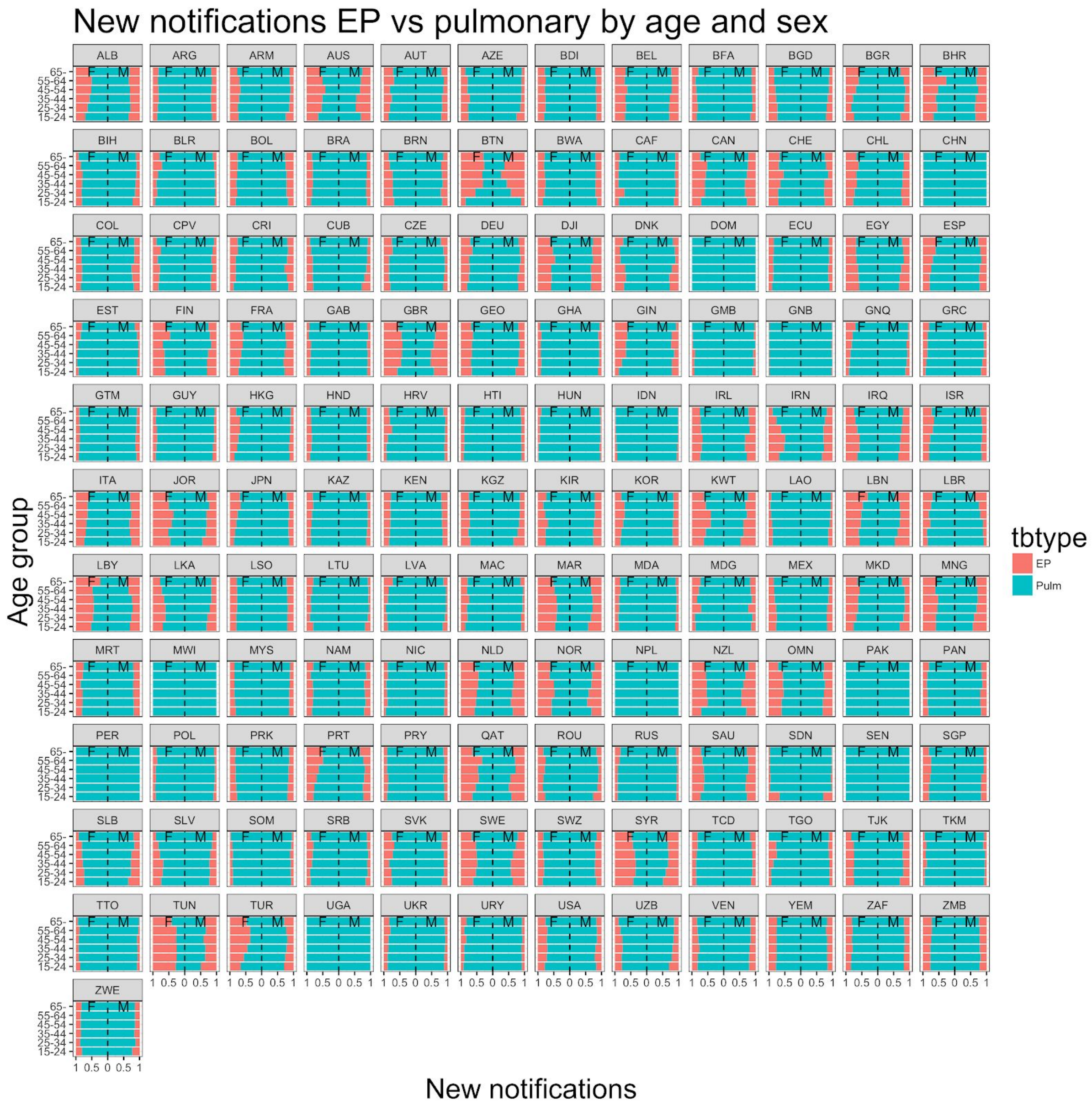

Figure 8: WHO data on the proportion of newly notified tuberculosis that is pulmonary (green) vs extrapulmonary (red) by age (y-axis), sex (left of dashed line=female; right of dashed line=male), and country (panel), aggregated over all years with complete data.

Other countries were assigned regional means of proportion pulmonary for each age and sex (see Figure 9), and these proportions were applied to notifications by age and sex to arrive at the number of pulmonary notifications to be followed up with HHCM in a given country.

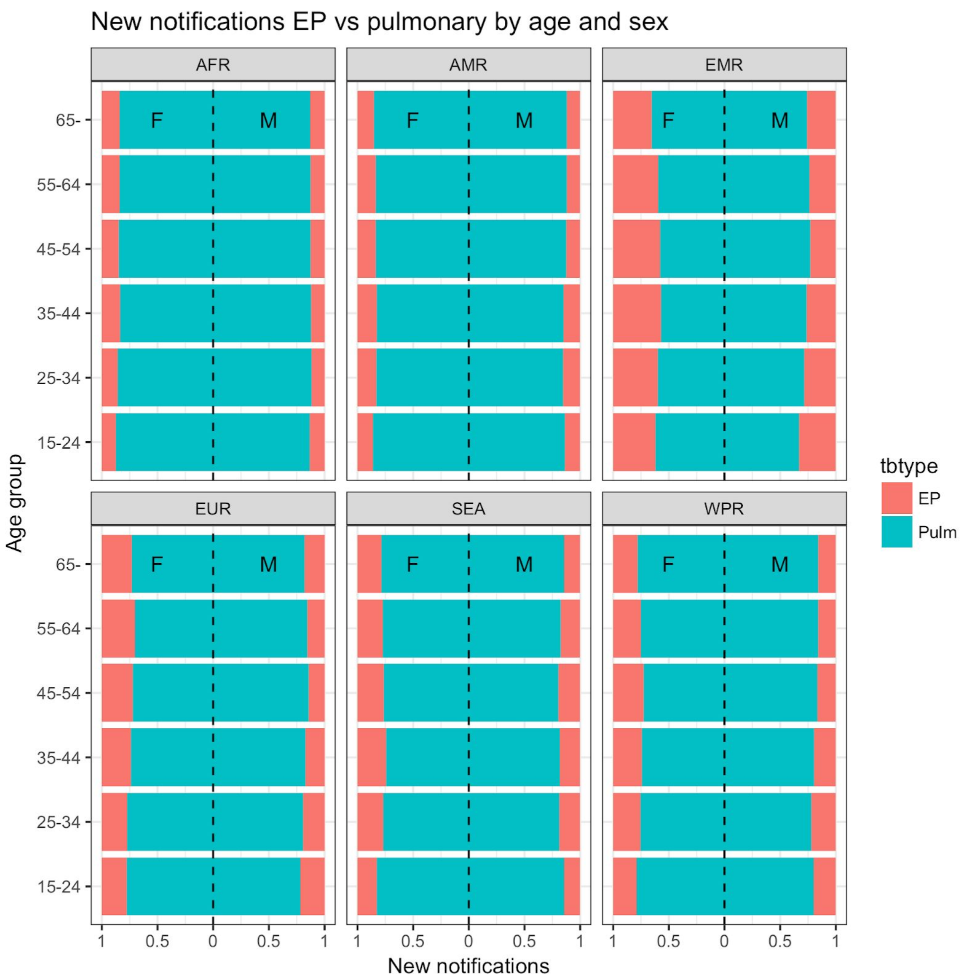

Figure 9: WHO data on the proportion of newly notified tuberculosis that is pulmonary (green) vs extrapulmonary (red) by age (y-axis), sex (left of dashed line=female; right of dashed line=male), and WHO region (panel), averaged over all years with complete data.

Decision tree model structure

The model was implemented as a decision tree using the open source R package *HEdtree* (https://github.com/petedodd/HEdtree). The logic of the decision tree is shown in Figure 10: boxes show intermediate and final outcomes beginning with a child household contact of a TB case of a given age and HIV/ART status. The labels on the arrows denote probabilities of following a particular branch in the tree; these probabilities may depend on the age and HIV/ART status of children. The model is specified within this framework in full on the repository at https://github.com/petedodd/PINT The model object automatically generated Figure 10 (which therefore serves as a way to assess the specified logic), and also generates functions that evaluate the means of quantities (e.g. incidence, prevalence, deaths, life-expectancy etc) over the tree for a given set of input parameters (sampled from the distributions specified below).

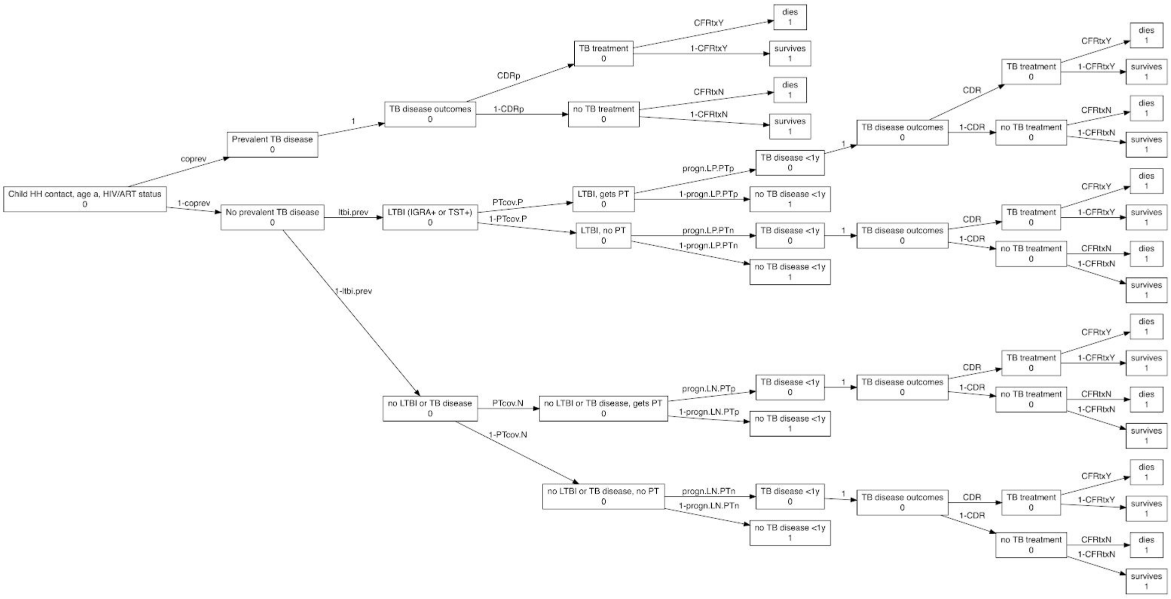

Figure 10: Model structure and transition probability names. Transition probability functions (which may depend on individual and intervention characteristics etc) are defined in model code. NB in the main article, we simplified this tree by removing subtrees with zero probability of being entered; here we include them to reflect how they were modelled and to locate all the parameters.

Modelling approach and evidence

In this section we describe the modelling logic and choices for particular aspects of the model in more detail.

Number of household contacts

The statistical models described in the previous section were used to generate 1,000 predictions for the mean number of cohabiting children age [0,5) and [5,15) by age and sex of adult, for each of 180 countries for which World Bank and WHO data were available (ISO3 codes: ABW, AFG, AGO, AIA, ALB, AND, ARE, ARG, ARM, ASM, ATG, AUS, AUT, AZE, BDI, BEL, BEN, BES, BFA, BGD, BGR, BHR, BHS, BIH, BLR, BLZ, BMU, BOL, BRA, BRB, BRN, BTN, BWA, CAF, CAN, CHE, CHL, CHN, CIV, CMR, COD, COG, COK, COL, COM, CPV, CRI, CUB, CUW, CYM, CYP, CZE, DEU, DJI, DMA, DNK, DOM, DZA, ECU, EGY, ERI, ESP, EST, ETH, FIN, FJI, FRA, FSM, GAB, GBR, GEO, GHA, GIN, GMB, GNB, GNQ, GRC, GRD, GRL, GTM, GUM, GUY, HKG, HND, HRV, HTI, HUN, IDN, IND, IRL, IRN, IRQ, ISL, ISR, ITA, JAM, JOR, JPN, KAZ, KEN, KGZ, KHM, KIR, KNA, KOR, KWT, LAO, LBN, LBR, LBY, LCA, LKA, LSO, LTU, LUX, LVA, MAC, MAR, MCO, MDA, MDG, MDV, MEX, MHL, MKD, MLI, MLT, MMR, MNE, MNG, MNP, MOZ, MRT, MSR, MUS, MWI, MYS, NAM, NCL, NER, NGA, NIC, NIU, NLD, NOR, NPL, NRU, NZL, OMN, PAK, PAN, PER, PHL, PLW, PNG, POL, PRI, PRK, PRT, PRY, PSE, PYF, QAT, ROU, RUS, RWA, SAU, SDN, SEN, SGP, SLB, SLE, SLV, SMR, SOM, SRB, SSD, STP, SUR, SVK, SVN, SWE, SWZ, SXM, SYC, SYR, TCA, TCD, TGO, THA, TJK, TKL, TKM, TLS, TON, TTO, TUN, TUR, TUV, TZA, UGA, UKR, URY, USA, UZB, VCT, VEN, VGB, VIR, VNM, VUT, WLF, WSM, YEM, ZAF, ZMB, ZWE). To extend this to the 217 countries in the WHO TB notification data, we used WHO regional averages of quantities from the 180 countries for the missing 47 countries.

For each of these countries, the WHO TB notification data stratified by age and sex were first adjusted by the proportion pulmonary for each group, then merged with the child household contact predictions (by age, sex and country) and the predicted number of total child TB household contacts in each country computed for each prediction. This results of this analysis were summarised by modelling the distribution of child household TB contacts in each country using separate log-normal distributions.

Prevalence and incidence in household contacts

The prevalence of active TB and latent TB infection (LTBI) in child household contacts of TB patients were based on the meta-analysis of Fox *et al.*,[1] sampling separately for children in age groups [0,5) and [5,15) years, using the data for low and middle-income countries and high-income countries separately. Co-prevalent children were excluded from LTBI and the resulting number of infections taken as the at risk group for progression to incident TB disease within one year. This model of progression to disease was that used in Dodd *et al.*[2] Since progression risks following infection in children have been based predominantly on TST status,[3] we used estimates of infection risk determined by TST to estimate the number at risk of progressing to disease.

The prevalence of disease is represented by the decision tree probability 'coprev' in Figure 10, which is a function of age and country income group, and depends on parameters 'coprev04', 'coprev514', 'coprev04hi' & 'coprev514hi' in Table 4 below. The prevalence of disease is represented by the decision tree probability 'ltbi.prev', which is a function of age and country income group, and depends on parameters 'LTBI04', 'LTBI514', 'LTBI04hi' & 'LTBI514hi' in Table 4 below.

Case detection

The overall case detection ratio (CDR) for children age [0,5) and [5,15) for each country can be calculated as the ratio of estimated incidence and notifications reported by WHO. We estimate country-specific beta distribution parameters to model this parameter in each age group from the WHO data. However, we are interested in children cohabiting with notified TB cases, for whom the CDR might reasonably be expected to be higher. At one extreme, it could be the case that every child notified with TB is from a household with a notified adult TB patient. Previous work modelling work for high-burden countries[2] suggested around 70% of TB incidence in children might occur in households with adult TB cases, implying the upper-bound CDR for cohabiting children would be higher by a factor of 1/0.7 = 1.4 (corresponding to assuming all notifications were from households with an index case [numerator], but only 70% of total child incidence occurs in this group [denominator]). To account for this, we therefore scaled the mean of each beta distribution by a uniform variable lying between 1 and 2 (i.e. $\sim U[1,2]$, with mean 1.5), truncating the mean at 1.

This probability, which depends on age and country, is denoted 'CDR' ('CDRp' is identical in this work) in the decision tree model shown in Figure 10.

HIV

The prevalence of HIV among notified TB cases, and the coverage of ART in TB/HIV cases was determined for each country using the WHO notification data (the denominator being those with test results). There are limited data to inform the detailed relationship between HIV/ART of adult TB

cases and cohabiting children. We used data from Martinez *et al.*[4] to parametrize the probability of a child of age [0,5) or [5,15) years cohabiting with a notified HIV-positive TB case being HIV-positive. We assumed the same ART coverage among HIV-positive cohabiting children as among HIV-positive adult TB notifications. We modelled the impact of HIV/ART status on individual TB risk using the rate ratios in Dodd *et al.*[5]

HIV prevalence in contacts enters the decision tree model in Figure 10 via the HIV & ART prevalence in the entry cohort (at the root compartment). The parameters governing household child HIV prevalence are 'HHhivprev04' & 'HHhivprev514' in Table 4 below.

Mortality, life expectancy and IPT

Mortality for each age group and by anti-TB treatment status, HIV-infection status, and ART-status is modelled as in Dodd *et al*.[6]

That is, country- and age-dependent CDRs determine the proportion of child TB cases receiving treatment (see section above). Survival is described by the decision tree probabilities 'CFRtxY' & 'CDRtxN' for with/without treatment, respectively. These are functions of age, and HIV/ART status and are governed by the parameters in Table 3 below, ie: 'ontxY', 'ontxO', 'hivartOR:mn', 'hivartOR:sg', 'notxY', 'notxO', 'notxHY', 'notxHO', 'notxHAY' & 'notxHAO'.

Life expectancy in the absence of TB is taken from the WHO Global Health Observatory for each country and the mean life expectancy for children age [0,5) and [5,15) years. We did not use a separate life-expectancy for HIV-positive children.

PT was assumed to reduce the risk of developing incident TB over a year. In children of unknown TST status, the pooled estimate for ages <15 years from the systematic review and meta-analysis of Ayieko et al[7] was used, i.e. risk ratio of 0.65 (0.47-0.89), which is a lower level of protection than in adults. Because protection from PT is normally observed to be stronger in those with positive TST, for children receiving PT as the result of a positive TST, we used a variance-weighted pooled risk ratio based on the 3 studies identified by Ayieko et al with only TST positive children, i.e. 0.35 (0.04-0.66). For children receiving PT because they were HIV-positive we used the risk ratio from the recent systematic review and meta-analysis of Zumza et al.[8] on PT in HIV-positive children, i.e. 0.31 (0.11-0.87). These risk ratios were were all modelled as log-normal distributions.

Thus decision tree model parameters 'prognLNPTp' and 'prognLNPTn' in Figure 10 are identically zero in this work. The age, country-latitude and BCG/HIV/ART-status dependent parameters describing progression 'prognLPPTp' & 'prognLPPTn' in the decision tree model, therefore depend on all the parameters in Table 2 for the Dodd et al 2014 incidence model[2] (ie 'hivpi', 'artp', 'pp1', 'pp2', 'pp3', 'pp4', 'pp5', 'pd1', 'pd2', 'pd3', 'pd4', 'pd5', 'dBCG', 'pBCG' & 'vBCG'), as well as the HIV-dependent risk ratios parameters for PT from Table 4 ('iptRRtstpos', 'iptRRhivpos') for the first of these transitions.

To bound the potential for household contact management (HHCM), we considered three idealized interventions with perfect coverage:

A. a base case where no HHCM occurs;
B. HHCM following WHO guidelines with complete coverage - all prevalent TB in children is found, all children under 5 years and all HIV-positive children under 15 years are given PT;
C. HHCM as in B, but additionally giving PT to all tuberculin skin test positive children age [5,15) years.

In the decision tree model in Figure 10, these interventions define the age and HIV-status dependent preventive therapy coverages 'PTcovN' & 'PTcovP' for LTBI test-negative and positive, respectively.

For each intervention, we calculated the number of households visited, the number of children screened for TB, the number of children identified with co-prevalent TB, the number of anti-tuberculosis treatments dispensed, the number of PT courses dispensed, the number of children developing incident TB, the number of deaths due to TB, and the expected number of life-years lived by children cohabiting with notified TB cases. We also calculated incremental measures of effort and effect between interventions B and C and the base case A.

Model parameters, distributions and evidence

The parameters in Table 2 pertain to the incidence model described in detail in Dodd et al 2014[2] and Dodd et al 2017[6] and their Appendices. The parameters in Table 3 pertain to the mortality model described in detail in Dodd et al 2017[6]. The parameters in Table 4 are specific to modelling HHCM. See above for discussion of parameters.

Table 2: Parameters relevant to TB incidence. (Dodd et al 2017,[5] Marais et al 2004,[3] Colditz et al 1995,[9] Bourdin Trunz et al 2006[10])

**Name**	**Distribution**	**Mean & quartiles**	**Description**	**Source**
hivpi	LN(2.066863,0.2800718)	7.900 (6.540 - 9.543)	IRR for TB incidence given HIV+/ART- (for individuals)	Dodd et al 2017
artp	LN(-1.203973,0.150482)	0.300 (0.271 - 0.332)	HR for TB incidence given HIV+/ART+ vs HIV+/ART-	Dodd et al 2017
pp1	B(1.5000,1.5000)	0.500 (0.298 - 0.702)	Probability of progression to TB, age <1	Marais et al 2004
pp2	B(1.2500,3.7500)	0.215 (0.108 - 0.360)	Probability of progression to TB, age [1,2)	Marais et al 2004
pp3	B(0.3300,6.2700)	0.016 (0.002 - 0.064)	Probability of progression to TB, age [2,5)	Marais et al 2004
pp4	B(0.1368,6.7032)	0.001 (0.000 - 0.013)	Probability of progression to TB, age [5,10)	Marais et al 2004
pp5	B(0.8700,4.9300)	0.110 (0.043 - 0.219)	Probability of progression to TB, age [10,15)	Marais et al 2004
pd1	B(1.5000,1.5000)	0.500 (0.298 - 0.702)	Probability of DTB if TB, age <1	Marais et al 2004
pd2	B(1.2500,3.7500)	0.215 (0.108 - 0.360)	Probability of DTB if TB, age [1,2)	Marais et al 2004
pd3	B(0.3300,6.2700)	0.016 (0.002 - 0.064)	Probability of DTB if TB, age [2,5)	Marais et al 2004
pd4	B(0.1368,6.7032)	0.001 (0.000 - 0.013)	Probability of DTB if TB, age [5,10)	Marais et al 2004
pd5	B(0.8700,4.9300)	0.110 (0.043 - 0.219)	Probability of DTB if TB, age [10,15)	Marais et al 2004
dBCG	B(1.25,2.5)	0.301 (0.155 - 0.484)	Protection of BCG against DTB	Colditz et al 1995, Bourdin Trunz et al 2006
pBCG	B(4,1)	0.841 (0.707 - 0.931)	Fraction of BCG DTB protection applying to PTB	Colditz et al 1995, Bourdin Trunz et al 2006
vBCG	0.41		Fraction of BCG efficacy lost from pole to equator	Colditz et al 1995

Table 3: Parameters relevant to TB mortality. (Jenkins et al 2017,[11] Dodd et al 2017[6])

Name	Distribution	Mean & quartiles	Description	Source
ontxY	LN(-3.963316,0.6457913)	0.019 (0.012 - 0.029)	CFR children <5 on TB treatment	Jenkins et al 2017
ontxO	LN(-4.828314,0.4817445)	0.008 (0.006 - 0.011)	CFR children 5-14 on TB treatment	Jenkins et al 2017
hivartOR:mn	MVN: [2.6375681, -0.5683867]		ORs of death on TB treatment, (OR HIV+ vs -) x (ART -/+): mean	Jenkins et al 2017, Dodd et al 2017
hivartOR:sg	MVN: [[0.2325509,-0.2325509],[-0.2325509,0.6367345]]		ORs of death on TB treatment, (OR HIV+ vs -) x (ART -/+): variance	Jenkins et al 2017, Dodd et al 2017
notxY	LN(-0.830113,0.08035318)	0.436 (0.413 - 0.460)	CFR children <5 without TB treatment	Jenkins et al 2017
notxO	LN(-1.903809,0.1285165)	0.149 (0.137 - 0.162)	CFR children 5-14 without TB treatment	Jenkins et al 2017
notxHY	B(77.13050,11.10817)	0.877 (0.852 - 0.899)	CFR children <5 without TB treatment (HIV+/ART-)	Dodd et al 2017
notxHO	B(19.59083,6.89700)	0.746 (0.686 - 0.800)	CFR children 5-14 without TB treatment (HIV+/ART-)	Dodd et al 2017
notxHAY	B(15.18683,12.87500)	0.542 (0.478 - 0.605)	CFR children <5 without TB treatment (HIV+/ART+)	Dodd et al 2017
notxHAO	B(10.43383,11.08417)	0.484 (0.412 - 0.558)	CFR children 5-14 without TB treatment (HIV+/ART+)	Dodd et al 2017

Table 4: Parameters relevant to HHCM. (Martinez et al 2018,[4] Fox et al 2013,[1] Ayieko et al 2014,[7] Zunza et al 2017[8])

Name	Distribution	Mean & quartiles	Description	Source
HHhivprev04	B(55,526)	0.094 (0.086 - 0.103)	Prevalence of HIV in child HH contacts of HIV+ index case	Martinez et al 2018
HHhivprev514	B(54,854)	0.059 (0.054 - 0.065)	Prevalence of HIV in child HH contacts of HIV+ index case	Martinez et al 2018
LTBI04	B(106.7330582,193.9234438)	0.355 (0.336 - 0.373)	LTBI coprevalence	Fox et al 2013
LTBI514	B(41.83776346,36.95275153)	0.531 (0.493 - 0.569)	LTBI coprevalence	Fox et al 2013
LTBI04hi	B(10.62231,54.54526)	0.160 (0.131 - 0.192)	LTBI coprevalence (high income)	Fox et al 2013
LTBI514hi	B(17.0386,75.56247)	0.182 (0.156 - 0.210)	LTBI coprevalence (high income)	Fox et al 2013
coprev04	B(7.057890378,63.52101	0.096 (0.074 -	TB coprevalence	Fox et al 2013

	341)	0.122)		
coprev514	B(2.449353527,26.70961703)	0.075 (0.046 - 0.112)	TB coprevalence	Fox et al 2013
coprev04hi	B(35.89632,727.8553)	0.047 (0.042 - 0.052)	TB coprevalence (high income)	Fox et al 2013
coprev514hi	B(10.82599,362.4841)	0.028 (0.023 - 0.034)	TB coprevalence (high income)	Fox et al 2013
iptRR	LN(-0.4307829,0.1616345)	0.650 (0.583 - 0.725)	RR for incident TB given IPT, age <15	Ayieko et al 2014
iptRRtstpos	LN(-1.049822,0.4004678)	0.350 (0.267 - 0.459)	RR for incident TB given IPT in TST+, age <15	Ayieko et al 2014
iptRRhivpos	LN(-1.171183,0.5127492)	0.310 (0.219 - 0.438)	RR for incident TB given IPT in HIV+, age <15	Zunza et al 2017

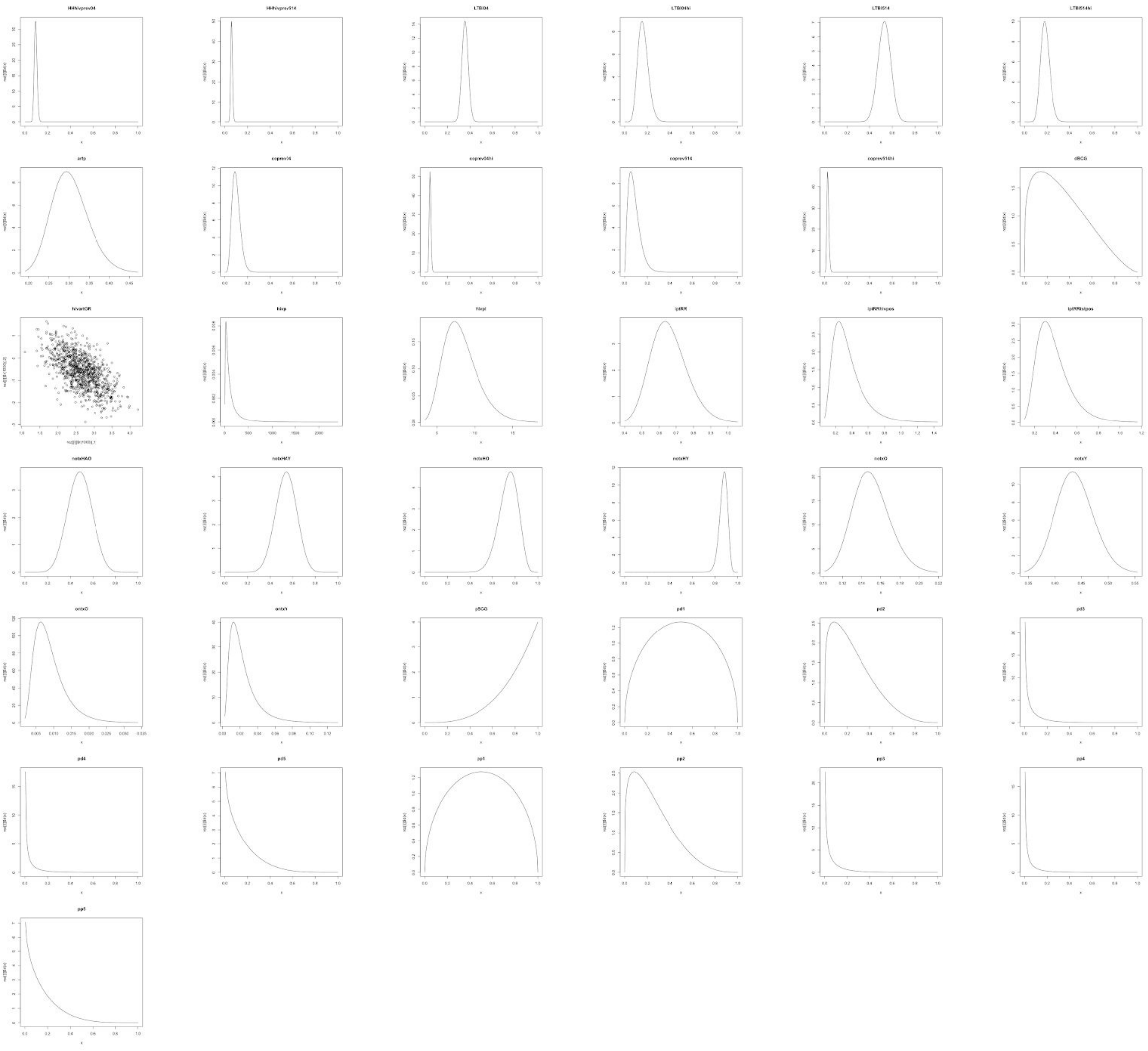

Figure 11: Probability distributions for all input parameters.

Figure 11 shows the probability density functions for all input parameters. Individual graphs of all distributions and a table of distribution means and quartiles are available at https://github.com/petedodd/PINT/test

Probabilistic sensitivity analysis

The distributions characterising the model input parameters were sampled from to generate 1,000 parameter sets. This dataset was replicated and parameters representing interventions modified before merging. This merged dataset was merged with a dataset of countries (including parameters characterising the mean and variance of the number of child household TB contacts for each country and in each age group). Model outputs for this joint dataset were calculated using the outcome functions determined by the *HEdtree* package and summaries of the results produced by intervention, and by country, region and globally.

Results

Regional and global results

Table 5: Additional results by region; standard deviation in brackets. (households = households visited; hhc = child household contacts screened; ATTprev = anti-TB treatment courses given to children coprevalent at index case notification; ATTinc = anti-TB treatment courses given to children incident subsequent to index case notification; IPT = (isoniazid) preventive therapy courses given; LTBI = latent tuberculosis infection; prevalent = coprevalent at index case notification; incidence = incident after index case notification; cases = coprevalence + incidence; deathprev = tuberculosis deaths in coprevalent children; deathinc = tuberculosis deaths in incident children; deaths; LE = years of life expectancy)

intervention	variable	Global	AFR	AMR	EMR	EUR	SEA	WPR	[0,5)	[5,15)
No HHCM	household	0 (0)	0 (0)	0 (0)	0 (0)	0 (0)	0 (0)	0 (0)	0 (0)	0 (0)
	hhc	0 (0)	0 (0)	0 (0)	0 (0)	0 (0)	0 (0)	0 (0)	0 (0)	0 (0)
	ATTprev	441,900 (86,710)	135,900 (17,040)	8,617 (1,521)	49,880 (28,440)	6,877 (1,550)	202,600 (80,000)	37,940 (12,720)	118,100 (15,780)	323,700 (85,100)
	ATTinc	152,400 (20,150)	50,460 (4,743)	2,971 (398)	16,860 (8,306)	2,090 (350)	67,440 (17,660)	12,560 (3,166)	45,020 (6,071)	107,400 (19,190)
	ATT	594,200 (89,930)	186,400 (17,880)	11,590 (1,595)	66,740 (34,040)	8,967 (1,648)	270,000 (81,540)	50,500 (13,100)	163,200 (18,200)	431,100 (87,830)
	IPT	0 (0)	0 (0)	0 (0)	0 (0)	0 (0)	0 (0)	0 (0)	0 (0)	0 (0)
	LTBI	3,535,000 (124,500)	1,064,000 (31,290)	87,210 (3,809)	395,400 (35,480)	71,300 (4,048)	1,439,000 (112,000)	477,700 (34,500)	886,800 (33,760)	2,648,000 (118,600)
	prevalent	739,200 (92,950)	223,800 (21,850)	17,850 (2,828)	81,220 (25,560)	14,980 (2,604)	301,500 (85,020)	99,920 (24,750)	278,300 (32,440)	461,000 (87,240)
	incidence	257,300 (23,420)	83,920 (6,274)	6,228 (742)	27,660 (6,703)	4,653 (605)	102,000 (20,700)	32,790 (6,174)	104,500 (13,320)	152,800 (19,070)
	cases	996,500 (94,090)	307,700 (22,350)	24,070 (2,879)	108,900 (26,040)	19,630 (2,635)	403,500 (85,810)	132,700 (25,280)	382,800 (34,500)	613,700 (87,150)
	deathprev	97,390 (12,920)	28,230 (2,795)	2,688 (388)	7,453 (4,243)	2,502 (371)	39,460 (10,810)	17,060 (4,308)	73,330 (11,160)	24,060 (5,765)
	deathinc	36,100 (4,937)	11,680 (1,148)	1,018 (138)	2,625 (1,340)	849 (130)	14,130 (4,340)	5,794 (1,382)	27,680 (4,579)	8,418 (1,630)
	deaths	133,500 (14,590)	39,910 (3,270)	3,706 (421)	10,080 (5,152)	3,351 (408)	53,580 (12,040)	22,850 (4,642)	101,000 (12,460)	32,480 (6,691)
	LE	526,200,000 (10,300,000)	145,800,000 (2,415,000)	14,240,000 (333,800)	59,890,000 (2,994,000)	11,900,000 (433,100)	218,300,000 (9,269,000)	76,040,000 (3,149,000)	182,900,000 (4,716,000)	343,400,000 (9,120,000)
Under 5 & HIV+ve	household	5,100,000 (0)	994,000 (0)	185,100 (0)	408,600 (0)	207,800 (0)	2,106,000 (0)	1,198,000 (0)	5,100,000 (0)	5,100,000 (0)
	hhc	8,258,000 (158,800)	2,487,000 (40,990)	206,600 (4,867)	919,200 (45,320)	178,500 (6,738)	3,344,000 (142,500)	1,123,000 (46,260)	2,789,000 (70,050)	5,469,000 (142,100)
	ATTprev	739,200 (92,950)	223,800 (21,850)	17,850 (2,828)	81,220 (25,560)	14,980 (2,604)	301,500 (85,020)	99,920 (24,750)	278,300 (32,440)	461,000 (87,240)
	ATTinc	123,200 (19,530)	38,550 (4,295)	2,360 (370)	11,620 (7,809)	1,662 (327)	59,040 (17,270)	10,020 (2,837)	17,110 (3,186)	106,100 (19,180)
	ATT	862,500 (93,240)	262,300 (21,990)	20,210 (2,823)	92,830 (26,910)	16,640 (2,613)	360,500 (85,200)	109,900 (24,850)	295,400 (32,550)	567,100 (87,350)
	IPT	2,543,000 (68,610)	805,100 (19,640)	60,840 (2,223)	272,100 (20,550)	58,530 (2,978)	1,012,000 (60,020)	333,800 (19,370)	2,511,000 (68,620)	31,900 (1,292)
	LTBI	3,535,000 (124,500)	1,064,000 (31,290)	87,210 (3,809)	395,400 (35,480)	71,300 (4,048)	1,439,000 (112,000)	477,700 (34,500)	886,800 (33,760)	2,648,000 (118,600)
	prevalent	739,200 (92,950)	223,800 (21,850)	17,850 (2,828)	81,220 (25,560)	14,980 (2,604)	301,500 (85,020)	99,920 (24,750)	278,300 (32,440)	461,000 (87,240)
	incidence	190,600 (20,610)	60,560 (5,459)	4,647 (659)	20,850 (5,927)	3,344 (511)	76,450 (18,170)	24,720 (5,497)	39,720 (7,403)	150,800 (19,060)
	cases	929,800 (93,380)	284,300 (22,100)	22,490 (2,851)	102,100 (25,820)	18,320 (2,623)	377,900 (85,380)	124,600 (25,070)	318,000 (33,130)	611,800 (87,150)
	deathprev	11,420 (2,053)	3,772 (543)	273 (58)	1,189 (572)	237 (61)	4,516 (1,867)	1,437 (502)	7,035 (1,703)	4,389 (1,119)

	deathinc	18,420 (2,988)	5,993 (682)	556 (91)	1,807 (1,163)	437 (78)	6,316 (2,460)	3,308 (886)	10,510 (2,433)	7,906 (1,624)
	deaths	29,840 (3,730)	9,766 (898)	828 (107)	2,996 (1,295)	674 (99)	10,830 (3,148)	4,745 (1,028)	17,550 (3,038)	12,290 (2,029)
	LE	533,200,000 (10,370,000)	147,700,000 (2,430,000)	14,450,000 (338,700)	60,360,000 (2,980,000)	12,090,000 (438,300)	221,300,000 (9,305,000)	77,370,000 (3,199,000)	188,600,000 (4,833,000)	344,700,000 (9,140,000)
**Under 5 & HIV+ve & TST+**	household	5,100,000 (0)	994,000 (0)	185,100 (0)	408,600 (0)	207,800 (0)	2,106,000 (0)	1,198,000 (0)	5,100,000 (0)	5,100,000 (0)
	hhc	8,258,000 (158,800)	2,487,000 (40,990)	206,600 (4,867)	919,200 (45,320)	178,500 (6,738)	3,344,000 (142,500)	1,123,000 (46,260)	2,789,000 (70,050)	5,469,000 (142,100)
	ATTprev	739,200 (92,950)	223,800 (21,850)	17,850 (2,828)	81,220 (25,560)	14,980 (2,604)	301,500 (85,020)	99,920 (24,750)	278,300 (32,440)	461,000 (87,240)
	ATTinc	57,900 (10,440)	19,210 (2,731)	1,125 (208)	6,521 (3,869)	785 (171)	25,480 (9,184)	4,785 (1,649)	17,110 (3,186)	40,790 (9,942)
	ATT	797,200 (93,110)	243,000 (21,950)	18,970 (2,831)	87,740 (25,900)	15,760 (2,605)	327,000 (84,950)	104,700 (24,790)	295,400 (32,550)	501,800 (87,220)
	IPT	5,174,000 (138,600)	1,579,000 (36,450)	126,800 (4,221)	571,400 (40,830)	110,200 (4,880)	2,092,000 (124,400)	694,300 (38,620)	2,511,000 (68,620)	2,663,000 (118,700)
	LTBI	3,535,000 (124,500)	1,064,000 (31,290)	87,210 (3,809)	395,400 (35,480)	71,300 (4,048)	1,439,000 (112,000)	477,700 (34,500)	886,800 (33,760)	2,648,000 (118,600)
	prevalent	739,200 (92,950)	223,800 (21,850)	17,850 (2,828)	81,220 (25,560)	14,980 (2,604)	301,500 (85,020)	99,920 (24,750)	278,300 (32,440)	461,000 (87,240)
	incidence	97,760 (12,660)	31,910 (3,623)	2,364 (413)	10,570 (3,729)	1,753 (323)	38,620 (11,000)	12,540 (3,209)	39,720 (7,403)	58,040 (10,300)
	cases	837,000 (93,370)	255,700 (22,040)	20,210 (2,851)	91,790 (25,650)	16,730 (2,608)	340,100 (85,150)	112,500 (24,890)	318,000 (33,130)	519,000 (87,220)
	deathprev	11,420 (2,053)	3,772 (543)	273 (58)	1,189 (572)	237 (61)	4,516 (1,867)	1,437 (502)	7,035 (1,703)	4,389 (1,119)
	deathinc	13,710 (2,573)	4,425 (576)	387 (75)	990 (572)	322 (68)	5,367 (2,294)	2,217 (715)	10,510 (2,433)	3,195 (725)
	deaths	25,130 (3,381)	8,198 (815)	659 (95)	2,179 (819)	559 (91)	9,883 (2,989)	3,654 (885)	17,550 (3,038)	7,584 (1,380)
	LE	533,500,000 (10,370,000)	147,800,000 (2,432,000)	14,460,000 (338,900)	60,410,000 (2,983,000)	12,100,000 (438,600)	221,400,000 (9,306,000)	77,440,000 (3,203,000)	188,600,000 (4,833,000)	344,900,000 (9,145,000)
**B-A**	household	5,100,000 (0)	994,000 (0)	185,100 (0)	408,600 (0)	207,800 (0)	2,106,000 (0)	1,198,000 (0)	5,100,000 (0)	5,100,000 (0)
	hhc	8,258,000 (158,800)	2,487,000 (40,990)	206,600 (4,867)	919,200 (45,320)	178,500 (6,738)	3,344,000 (142,500)	1,123,000 (46,260)	2,789,000 (70,050)	5,469,000 (142,100)
	ATTprev	297,400 (46,710)	87,850 (10,960)	9,229 (1,693)	31,330 (26,250)	8,102 (1,542)	98,880 (30,120)	61,980 (19,210)	160,100 (24,480)	137,300 (38,170)
	ATTinc	-29,130 (4,294)	-11,910 (1,599)	-611 (110)	-5,242 (2,433)	-428 (102)	-8,404 (2,942)	-2,539 (1,170)	-27,900 (4,293)	-1,227 (152)
	ATT	268,300 (47,140)	75,940 (11,190)	8,618 (1,698)	26,090 (26,530)	7,674 (1,552)	90,480 (30,660)	59,450 (19,280)	132,200 (25,580)	136,000 (38,170)
	IPT	2,543,000 (68,610)	805,100 (19,640)	60,840 (2,223)	272,100 (20,550)	58,530 (2,978)	1,012,000 (60,020)	333,800 (19,370)	2,511,000 (68,620)	31,900 (1,292)
	LTBI	0 (0)	0 (0)	0 (0)	0 (0)	0 (0)	0 (0)	0 (0)	0 (0)	0 (0)
	prevalent	0 (0)	0 (0)	0 (0)	0 (0)	0 (0)	0 (0)	0 (0)	0 (0)	0 (0)
	incidence	-66,700 (9,889)	-23,360 (2,633)	-1,581 (274)	-6,807 (2,679)	-1,309 (274)	-25,580 (8,835)	-8,063 (2,413)	-64,800 (9,886)	-1,906 (211)
	cases	-66,700 (9,889)	-23,360 (2,633)	-1,581 (274)	-6,807 (2,679)	-1,309 (274)	-25,580 (8,835)	-8,063 (2,413)	-64,800 (9,886)	-1,906 (211)
	deathprev	-85,960 (12,250)	-24,460 (2,583)	-2,415 (358)	-6,264 (4,126)	-2,265 (344)	-34,940 (10,160)	-15,620 (4,081)	-66,290 (10,570)	-19,670 (5,479)
	deathinc	-17,680 (3,363)	-5,689 (751)	-462 (87)	-818 (507)	-412 (86)	-7,813 (3,104)	-2,486 (905)	-17,170 (3,361)	-513 (70)
	deaths	-103,600 (12,930)	-30,150 (2,850)	-2,877 (373)	-7,082 (4,296)	-2,677 (363)	-42,750 (10,730)	-18,110 (4,232)	-83,460 (11,330)	-20,180 (5,483)
	LE	7,006,000 (892,100)	1,827,000 (172,300)	210,300 (27,050)	472,700 (283,600)	187,100 (24,900)	2,983,000 (744,500)	1,326,000 (309,200)	5,724,000 (790,700)	1,282,000 (358,200)
**C-A**	household	5,100,000 (0)	994,000 (0)	185,100 (0)	408,600 (0)	207,800 (0)	2,106,000 (0)	1,198,000 (0)	5,100,000 (0)	5,100,000 (0)
	hhc	8,258,000 (158,800)	2,487,000 (40,990)	206,600 (4,867)	919,200 (45,320)	178,500 (6,738)	3,344,000 (142,500)	1,123,000 (46,260)	2,789,000 (70,050)	5,469,000 (142,100)
	ATTprev	297,400 (46,710)	87,850 (10,960)	9,229 (1,693)	31,330 (26,250)	8,102 (1,542)	98,880 (30,120)	61,980 (19,210)	160,100 (24,480)	137,300 (38,170)
	ATTinc	-94,470 (14,680)	-31,240 (3,267)	-1,846 (283)	-10,340 (5,409)	-1,305 (251)	-41,970 (13,160)	-7,774 (2,310)	-27,900 (4,293)	-66,570 (14,050)
	ATT	202,900 (51,940)	56,600 (11,890)	7,383 (1,761)	21,000 (29,720)	6,797 (1,615)	56,920 (34,660)	54,210 (19,390)	132,200 (25,580)	70,690 (43,960)

	IPT	5,174,000 (138,600)	1,579,000 (36,450)	126,800 (4,221)	571,400 (40,830)	110,200 (4,880)	2,092,000 (124,400)	694,300 (38,620)	2,511,000 (68,620)	2,663,000 (118,700)
	LTBI	0 (0)	0 (0)	0 (0)	0 (0)	0 (0)	0 (0)	0 (0)	0 (0)	0 (0)
	prevalent	0 (0)	0 (0)	0 (0)	0 (0)	0 (0)	0 (0)	0 (0)	0 (0)	0 (0)
	incidence	-159,500 (17,600)	-52,000 (4,564)	-3,863 (536)	-17,090 (4,895)	-2,900 (452)	-63,400 (15,770)	-20,240 (4,561)	-64,800 (9,886)	-94,710 (14,350)
	cases	-159,500 (17,600)	-52,000 (4,564)	-3,863 (536)	-17,090 (4,895)	-2,900 (452)	-63,400 (15,770)	-20,240 (4,561)	-64,800 (9,886)	-94,710 (14,350)
	deathprev	-85,960 (12,250)	-24,460 (2,583)	-2,415 (358)	-6,264 (4,126)	-2,265 (344)	-34,940 (10,160)	-15,620 (4,081)	-66,290 (10,570)	-19,670 (5,479)
	deathinc	-22,390 (3,578)	-7,256 (826)	-631 (97)	-1,635 (892)	-527 (94)	-8,763 (3,175)	-3,577 (1,021)	-17,170 (3,361)	-5,223 (1,091)
	deaths	-108,400 (13,230)	-31,710 (2,885)	-3,046 (376)	-7,899 (4,691)	-2,793 (367)	-43,700 (10,870)	-19,200 (4,281)	-83,460 (11,330)	-24,900 (6,059)
	LE	7,305,000 (910,200)	1,918,000 (174,300)	221,900 (27,260)	525,300 (308,900)	194,600 (25,140)	3,045,000 (752,700)	1,401,000 (312,600)	5,724,000 (790,700)	1,581,000 (395,000)

Supplementary Figure

The regional and country share of deaths preventable by HHCM is shown in Figure 12.

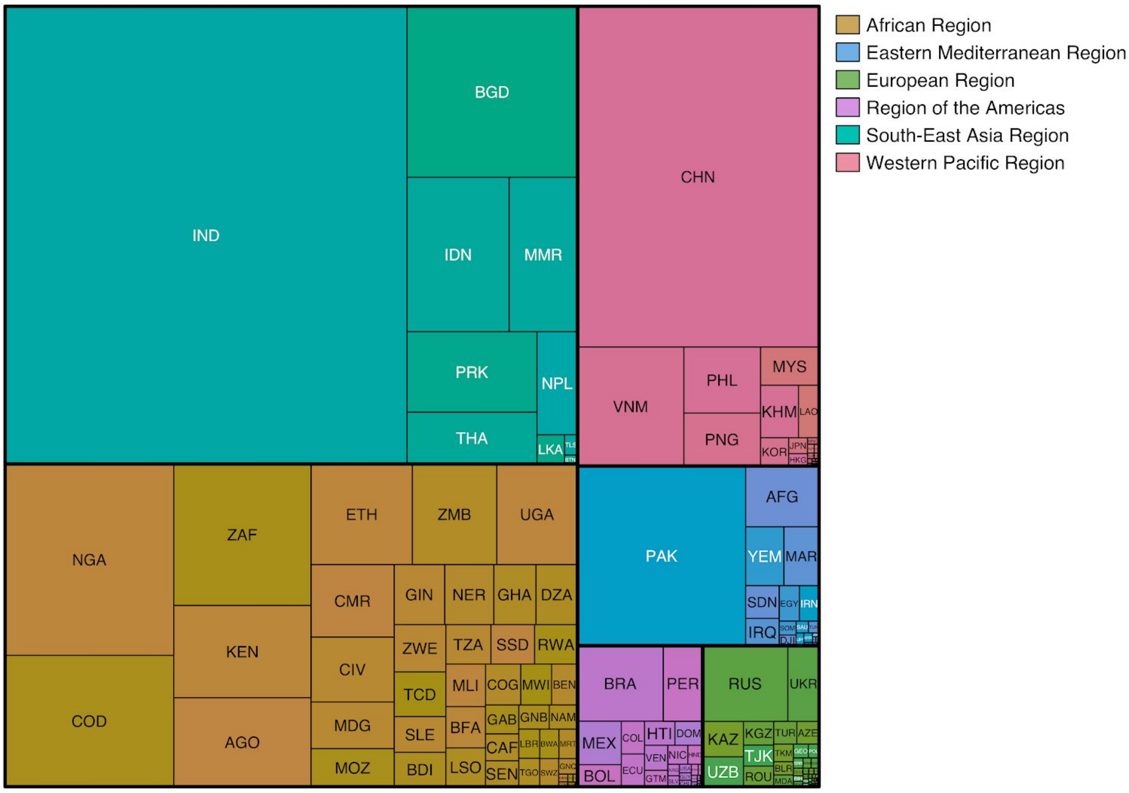

Figure 12: Number of preventable child tuberculosis deaths using household contact tracing by country and World Health Organization and country/territory (specified by ISO 3166-1 alpha-3 codes). The area of squares is proportional to deaths averted by moving from no household contact tracing (intervention A) to screening all household contacts under 15 years old and offering preventive therapy to children under 5 years old and HIV-infected children under 15 years old.

Availability of data and analysis code

All analyses were performed in R (version 3.3.2).

With the exception of the raw DHS data (sharing would contravene the data sharing agreement), all data and code are available at the GitHub repository: https://github.com/petedodd/PINT

All packages used are open source and either available from the Comprehensive R Archive Network (CRAN) or have their GitHub repositories linked (from which they can be installed using the *devtools* package).

References

1 Fox GJ, Barry SE, Britton WJ, Marks GB. Contact investigation for tuberculosis: a systematic review and meta-analysis. *Eur Respir J* 2012; **41**: 140–56.

2 Dodd PJ, Gardiner E, Coghlan R, Seddon JA. Burden of childhood tuberculosis in 22 high-burden countries: a mathematical modelling study. *Lancet Glob Health* 2014; **2**: e453–9.

3 Marais BJ, Gie RP, Schaaf HS, *et al.* The natural history of childhood intra-thoracic tuberculosis: a critical review of literature from the pre-chemotherapy era. *Int J Tuberc Lung Dis* 2004; **8**: 392–402.

4 Effectiveness of WHO's pragmatic screening algorithm for child contacts of tuberculosis cases in resource-constrained settings: a prospective cohort study in Uganda. *The Lancet Respiratory Medicine* 2017; published online Dec 19. DOI:10.1016/S2213-2600(17)30497-6.

5 Dodd PJ, Prendergast AJ, Beecroft C, Kampmann B, Seddon JA. The impact of HIV and antiretroviral therapy on TB risk in children: a systematic review and meta-analysis. *Thorax* 2017; **72**: 559–75.

6 Dodd PJ, Yuen CM, Sismanidis C, Seddon JA, Jenkins HE. The global burden of tuberculosis mortality in children: a mathematical modelling study. *Lancet Glob Health* 2017; **5**: e898–906.

7 Ayieko J, Abuogi L, Simchowitz B, Bukusi EA, Smith AH, Reingold A. Efficacy of isoniazid prophylactic therapy in prevention of tuberculosis in children: a meta–analysis. *BMC Infect Dis* 2014; **14**: 91.

8 Zunza M, Gray DM, Young T, Cotton M, Zar HJ. Isoniazid for preventing tuberculosis in HIV-infected children. *Cochrane Database Syst Rev* 2017; **8**: CD006418.

9 Colditz GA, Brewer TF, Berkey CS, *et al.* Efficacy of BCG vaccine in the prevention of tuberculosis. Meta-analysis of the published literature. *JAMA* 1994; **271**: 698–702.

10 Bourdin Trunz B, Fine P, Dye C. Effect of BCG vaccination on childhood tuberculous meningitis and miliary tuberculosis worldwide: a meta-analysis and assessment of cost-effectiveness. *Lancet* 2006; **367**: 1173–80.

11 Jenkins HE, Yuen CM, Rodriguez CA, *et al.* Mortality in children diagnosed with tuberculosis: a systematic review and meta-analysis. *Lancet Infect Dis* 2017; **17**: 285–95.
